# Socioeconomic and Demographic Risk Factors for SARS-CoV-2 Seropositivity Among Healthcare Workers in a UK Hospital: A Prospective Cohort Study

**DOI:** 10.1093/cid/ciad522

**Published:** 2023-08-30

**Authors:** Tanya Lam, Anja Saso, Arturo Torres Ortiz, James Hatcher, Marc Woodman, Shruthi Chandran, Rosie Thistlethwayte, Timothy Best, Marina Johnson, Helen Wagstaffe, Annabelle Mai, Matthew Buckland, Kimberly Gilmour, David Goldblatt, Louis Grandjean, COVID-19 Staff Testing of Antibody Responses (Co-STARs) Study Team, Dorcas Mirambe-Korsah, Fernanda Fenn Torrente, Jakub Wyszynski, Victoria Gander, Amy Leonard, Louise Myers, Aimee Vallot, Camille Paillas, Rose Fitzgerald, Adam Twigg, Rabia Manaf, Lois Gibbons, Hollie Powell, Richard Nar-Dorh, Ally Gray, Elias Fernandez, Aline Minja, Emily Beech, Waffa Girshab, Pei Shi Chia, Kate Webb, Malti Nakrani, Kim Gardiner, Valerija Karaluka, Karen Ryan, Dorothy Lee, Katie Groves, Hamad Khan, Shamime Nsubuga, Olivia Rosie-Wilkinson, Julia Spires, Nuria Sanchez-Clemente, Sapriya Kaur, Natasha Carroll, Jemma Efford, Gabriel Bredin, Celma Marisa Dos Santos Domingues, Sophie Foxall, Helen Ashton, Abbey Afzal, Sally Mainland, Kate Crumpler, Lucinda Dawson, Claire Smith, Maria Tabbu, Laura Chiverton, Jade Sugars, Jordan Mooney, Dorothy Chikusu, Fariba Tahami, Baratth Samy, Shomona Begum, Dhimple Patel, Philippa Wiltshire, Annie Susay, Anna Ryan, Luke Lancaster, Kavita Thind, Kate Speller, Rachel Sterling, Connor Tugulu, Sandhya Ghurburrun, Steffi Gray, Joy Mugas, Moe Kishma, Kathleen Akpokomua, Sophie White, Eleana Pieri, Sabina Shamsad, Demi Alexandrou, Odera Aguele, Katherine Miles, Anamika Jain, Subishma Gautam, Oliver Simms, Rachel Goff, Zarif Shams, Tinya Chirinda, Aaliya Nur, Tarekur Rahman

**Affiliations:** Department of Infectious Diseases, Great Ormond Street Hospital, London, United Kingdom; Department of Infectious Diseases, Great Ormond Street Hospital, London, United Kingdom; Department of Tropical and Infectious Diseases, London School of Hygiene and Tropical Medicine, London, United Kingdom; Medical Research Council Gambia at London School of Hygiene and Tropical Medicine, Fajara, The Gambia; Department of Infectious Diseases, Imperial College London, London, United Kingdom; Department of Infection, Immunity and Inflammation, Institute of Child Health, University College London, London, United Kingdom; Department of Microbiology, Great Ormond Street Hospital, London, United Kingdom; Department of Infection, Immunity and Inflammation, Institute of Child Health, University College London, London, United Kingdom; Department of Infection, Immunity and Inflammation, Institute of Child Health, University College London, London, United Kingdom; Management, Great Ormond Street Hospital, London, United Kingdom; Department of Microbiology, Great Ormond Street Hospital, London, United Kingdom; Department of Infection, Immunity and Inflammation, Institute of Child Health, University College London, London, United Kingdom; Department of Infection, Immunity and Inflammation, Institute of Child Health, University College London, London, United Kingdom; Clinical Immunology, Camelia Botnar Laboratories, Great Ormond Street Hospital, London, United Kingdom; Clinical Immunology, Camelia Botnar Laboratories, Great Ormond Street Hospital, London, United Kingdom; Clinical Immunology, Camelia Botnar Laboratories, Great Ormond Street Hospital, London, United Kingdom; Department of Infection, Immunity and Inflammation, Institute of Child Health, University College London, London, United Kingdom; Department of Infection, Immunity and Inflammation, Institute of Child Health, University College London, London, United Kingdom

**Keywords:** SARS-CoV-2, risk factors, healthcare workers, socioeconomic status, ethnicity

## Abstract

**Background:**

To protect healthcare workers (HCWs) from the consequences of disease due to severe acute respiratory syndrome coronavirus 2 (SARS-CoV-2), it is necessary to understand the risk factors that drive exposure and infection within hospitals. Insufficient consideration of key socioeconomic variables is a limitation of existing studies that can lead to bias and residual confounding of proposed risk factors for infection.

**Methods:**

The Co-STARs study prospectively enrolled 3679 HCWs between April 2020 and September 2020. We used multivariate logistic regression to comprehensively characterize the demographic, occupational, socioeconomic, and environmental risk factors for SARS-CoV-2 seropositivity.

**Results:**

After adjusting for key confounders, relative household overcrowding (odds ratio [OR], 1.4 [95% confidence interval {CI}, 1.1–1.9]; *P* = .006), Black, Black British, Caribbean, or African ethnicity (OR, 1.7 [95% CI, 1.2–2.3]; *P* = .003), increasing age (ages 50–60 years: OR, 1.8 [95% CI, 1.3–2.4]; *P* < .001), lack of access to sick pay (OR, 1.8 [95% CI, 1.3–2.4]; *P* < .001).

**Conclusions:**

Socioeconomic and demographic factors outside the hospital were the main drivers of infection and exposure to SARS-CoV-2 during the first wave of the pandemic in an urban pediatric referral hospital. Overcrowding and out-of-hospital SARS-CoV-2 contact are less amenable to intervention. However, lack of access to sick pay among externally contracted staff is more easily rectifiable. Our findings suggest that providing easier access to sick pay would lead to a decrease in SARS-CoV-2 transmission and potentially that of other infectious diseases in hospital settings.

**Clinical Trials Registration:**

NCT04380896.

## Teaser

This London-based prospective cohort study demonstrated an increased risk of SARS-CoV-2 seropositivity in healthcare workers reporting difficulty accessing sick leave, living in overcrowded housing, and/or of Black ethnicity, highlighting the effect of structural and ethnic inequality during the COVID-19 pandemic.

## Graphical Abstract

**Graphical Abstract:**
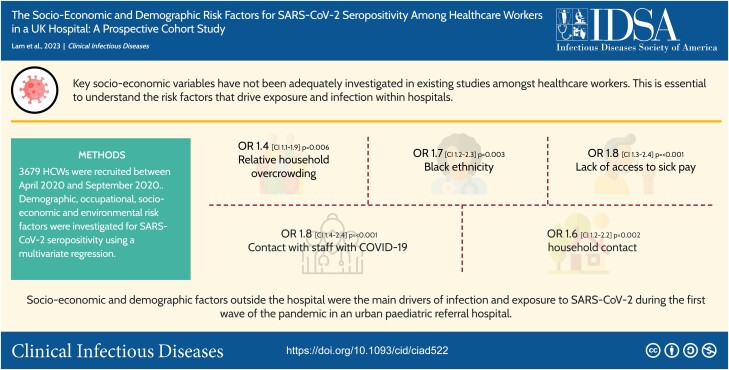
This graphical abstract is also available at Tidbit: https://tidbitapp.io/tidbits/the-socio-economic-and-demographic-risk-factors-for-sars-cov-2-seropositivity-among-healthcare-workers-in-a-uk-hospital-a-prospective-cohort-study/update

The coronavirus disease 2019 (COVID-19) pandemic placed unprecedented pressure on healthcare systems globally. Healthcare workers (HCWs) remained at the forefront of the pandemic during the first wave of infections, while nonessential workers were placed under stringent lockdown measures. Infection rates of COVID-19 in HCWs were higher than in the general population during this period, both in the United Kingdom (UK) and internationally [1–4]. Numerous studies have attempted to identify risk factors for HCW acquisition of COVID-19 with a view to creating safer working environments for staff and limiting the spread of healthcare-associated COVID-19 [5, 6].

Existing published risk factors for HCW exposure and infection vary widely [5–10]. There is considerable heterogeneity in clinical, demographic, occupational, and environmental variables recorded between studies [5, 11], limiting our understanding of specific risks and key confounders. For example, several international studies have identified that cleaners and hospital porters are those most at risk of exposure and infection [6, 7, 12, 13], but most studies have failed to include potential socioeconomic confounders of this association. Severe acute respiratory syndrome coronavirus 2 (SARS-CoV-2) has been detected in air samples in hospital rooms, on surfaces, and on shared staff equipment [14, 15], potentially exposing non-patient-facing staff—particularly cleaners and porters—to SARS-CoV-2. However, the Office for National Statistics (ONS) does not classify cleaners and hospital porters among the highest-exposure occupations [1]. This divergence suggests that local variability and residual confounding for SARS-CoV-2 acquisition may explain the association rather than the occupation itself. There are several published studies of SARS-CoV-2 seroprevalence in pediatric hospitals, but none has investigated ethnicity or socioeconomic variables as risk factors for SARS-CoV-2 exposure and infection [2–4, 16–18].

To improve our understanding of the underlying socioeconomic and demographic determinants of SARS-CoV-2 infection and exposure, we undertook a prospective cohort study of risk factors for SARS-CoV-2 seropositivity in a tertiary London pediatric hospital during the first wave of the pandemic.

## METHODS

### Study Setting, Design, and Participants

The COVID-19 Staff Testing of Antibody Responses (Co-STARs) project was a single-center prospective cohort study evaluating antibody responses to COVID-19 in HCWs at Great Ormond Street Hospital (GOSH). It was conducted between April 2020 and September 2020 during the first wave of the COVID pandemic [19, 20]. Both clinical and nonclinical hospital staff ≥18 years were invited to participate. To ensure equity of access to the study, face-to-face active recruitment was used for hospital staff on external contracts without National Health Service (NHS) Trust email addresses, who were more difficult to contact. These staff members included cleaners, porters, and catering staff. Participants were excluded if they had significant immunosuppression, recent administration of blood products (including immunoglobulins or convalescent sera) since September 2019, and persistent symptoms of SARS-CoV-2 infection at the time or within 21 days of recruitment. All participants signed an informed consent form, and the study was approved by the UK NHS Health Research Authority and registered at ClinicalTrials.gov (NCT04380896) [20].

### Data Collection

Blood samples were taken at baseline and at each follow-up visit for anti-SARS-CoV-2 immunoglobulin G serology using previously published methods [19, 20]. At the recruitment visit, participants also undertook a comprehensive, standardized online questionnaire (Supplementary Appendix  *C*). This included sociodemographic factors including self-assigned ethnicity [21]; details of previous exposure to SARS-CoV-2; symptomatic episodes consistent with COVID-19 with any subsequent complications; previous SARS-CoV-2 diagnostic test results; occupation and medical history; and a comprehensive assessment of risk factors for exposure, susceptibility to infection, and severe disease [20].

### Follow-up Appointments

All seropositive participants attended monthly follow-up visits for repeat antibody testing up to 250 days after the date of infection. Seronegative participants were followed up every 6 months. At each follow-up appointment, participants completed a shortened version of the baseline questionnaire, focusing on any significant changes since the last visit, including recurrent SARS-CoV-2 exposure and/or COVID-19 symptoms.

### Statistical Analysis

The risk of SARS-CoV-2 exposure or infection was estimated by fitting a logistic regression model using seropositivity for SARS-CoV-2 as the binary dependent outcome variable. Demographic (age, sex, and ethnicity), occupational (occupation, income, and working conditions), socioeconomic, environmental, and SARS-CoV-2 exposure factors were included as model predictors. Variables were chosen due to clinical and epidemiological relevance. As no statistical difference in seropositivity was estimated between White British, White Other, and White Irish ethnicities, these variables were merged into a single variable “White.” Univariate and multivariate logistic regression models were used to estimate odds ratios (ORs). Univariate models were fitted for each variable independently, while a multivariate regression was performed excluding variables that had a proportion of missing values >30%. Similarly, univariate and multivariate logistic regression models were performed to estimate the relationship between SARS-CoV-2 seropositivity and self-reported symptoms. Collinearity was assessed by calculating the variance inflation factor (VIF) for all variables selected in the multivariate model. All VIF values ranged between 1 and 2, suggesting that no collinearity was detected in our model, and therefore no variable was removed.

The impact of socioeconomic deprivation on the risk of SARS-CoV-2 seropositivity was included in the model by linking the postal code metadata to area deprivation using the index of multiple deprivation as reported by the UK Ministry of Housing, Communities and Local Government [22].

All analyses were performed using R software [23].

The Supplementary Material includes the study protocol (for study flowcharts see Supplementary Appendix A, for schedule of procedures see Supplementary Appendix B), power calculations (Supplementary Figures 1 and 2), detailed laboratory methodology, the questionnaires used for data collection (Supplementary Appendix C), and sites of international collaboration (Supplementary Appendix D).

## RESULTS

A total of 3646 staff members were recruited out of a total 5755 employees at GOSH (63.3%). Of the total number of staff approached for recruitment, <1% declined to participate. There were 53 confirmed inpatient cases with COVID-19 diagnosed by polymerase chain reaction on nasopharyngeal swabs during the study period. As shown in Table 1, 24% (712/3646) of the participants were categorized as seropositive, defined as presenting a SARS-CoV-2–positive test at any point during the study period. The majority of the participants were female (77% [2801/3646]) and White (54% [1951/3646]). Most participants self-reported symptoms (64%), but 1.3% (48/3646) sought medical attention and only 11 of them (0.3%) required hospitalization (Table 1).

**Table 1.** Demographics of Study Participants

Characteristic	Total	Seropositive
Total participants	3646 (100)	712 (24.26)
Demographic characteristics		
Age, y		
17–30	1062 (29.12)	172 (16.19)
30–40	1183 (32.44)	213 (18.00)
40–50	731 (20.05)	170 (23.25)
50–60	513 (14.07)	122 (23.78)
60–80	156 (4.27)	35 (22.43)
Sex		
Male	808 (22.16)	167 (20.67)
Female	2801 (76.82)	543 (19.38)
Undetermined	30 (0.82)	0 (0)
Ethnicity		
White	1951 (53.51)	410 (21.01)
Any other	64 (1.75)	15 (23.44)
Arab	31 (0.85)	8 (25.80)
South Asian	416 (11.41)	110 (26.44)
Black	259 (7.10)	95 (36.68)
Chinese	49 (1.34)	11 (22.45)
Mixed	95 (2.6)	24 (25.26)
Occupation		
AHPs	814 (22.32)	155 (19.04)
Cleaning/Catering/Porters	190 (5.21)	71 (37.36)
Doctor	588 (16.12)	96 (16.32)
ICT	44 (1.21)	13 (29.55)
Manager	213 (5.8)	44 (20.66)
Nurse	1258 (34.50)	233 (18.52)
Other	237 (6.50)	56 (23.63)
Scientist	299 (8.20)	44 (14.72)
Symptoms and severity		
Asymptomatic	1316 (36.09)	266 (20.21)
Abnormal smell sensation	425 (11.66)	257 (60.47)
Abnormal taste sensation	477 (13.08)	276 (57.86)
Admitted to hospital	11 (0.3)	5 (45.45)
Altered conscious state	11 (0.30)	5 (45.45)
Attended hospital	48 (1.32)	16 (33.33)
Chills	162 (4.44)	56 (34.57)
Conjunctivitis	21 (0.58)	8 (38.1)
Cough	1003 (27.51)	313 (31.21)
Diarrhea	301 (8.26)	113 (37.54)
Extreme fatigue	700 (19.20)	267 (38.14)
Fever (temperature *>*38°C)	649 (17.80)	244 (37.6)
Headache	254 (6.97)	91 (35.83)
Loss of appetite	148 (4.06)	58 (39.19)
Muscle pain	796 (21.83)	300 (37.69)
Nose bleed	19 (0.52)	8 (42.11)
Runny nose	597 (16.37)	176 (29.48)
Shortness of breath	555 (15.22)	174 (31.35)
Vomiting	80 (2.19)	27 (33.75)
Wheeze	318 (8.72)	96 (30.19)

Data are presented as No. (%).

Abbreviations: AHPs, allied health professionals; ICT, Information Computing Technology.

### SARS-CoV-2 Risk Factors for HCWs

#### Demographic Risks of SARS-CoV-2 Infection or Exposure

**Figure 1.**
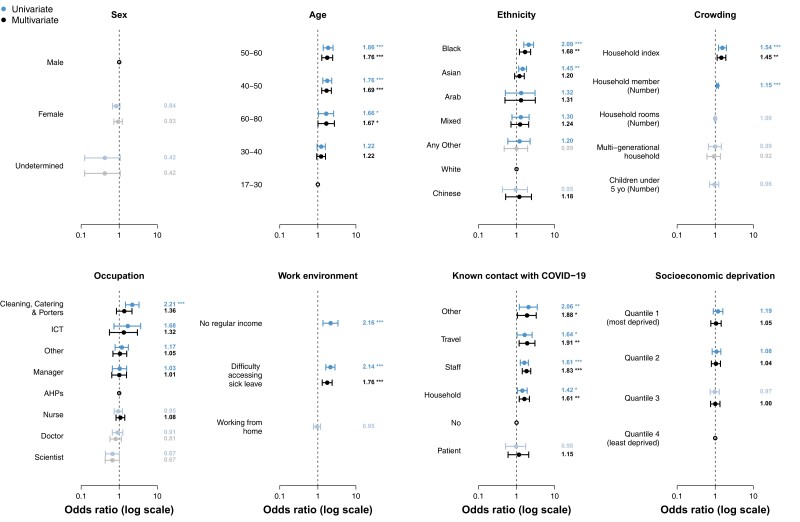
Risk factors for severe acute respiratory syndrome coronavirus 2 (SARS-CoV-2) seropositivity among healthcare workers (HCWs). Risk of SARS-CoV-2 seropositivity among HCWs was estimated using univariate and multivariate logistic regression model. Points show the best estimate of the odds ratio (OR), while error bars represent the 95% confidence interval (CI) for the estimate OR. Bold indicates whether the OR is higher than the reference group (bold) or lower (gray). Statistical significance of the estimated ORs is presented next to the CI bars. *P < .05; **p<0.01; ***P < .001. Abbreviations: AHPs, allied health professionals; COVID-19, coronavirus disease 2019; ICT, Information Computing Technology.

No difference in SARS-CoV-2 seropositivity was observed between male or female staff. Higher rates of seropositivity were seen in those aged 40–60 years, which remained significant in multivariate analysis. On univariate analysis, Black and South Asian ethnicity were associated with higher rates of seropositivity (40.0% and 26.4%, respectively). However, on multivariate analysis, South Asian ethnicity was no longer significant, whereas Black ethnicity remained significant (OR, 1.7 [95% confidence interval {CI}, 1.2–2.3]; *P* = .003) (Figure 1 and Table 2)

**Table 2.** Association of Risk Factors With COVID-19 Seropositivity

Characteristic	Total, No.	Seropositive, No.	Univariate		Multivariate	
			OR (95% CI)	*P* Value	OR (95% CI)	*P* Value
Demographic characteristics						
Age, y						
17–30	719	142	1.0 (reference)	…	1.0 (reference)	…
30–40	752	174	1.2 (1.0–1.6)	.114	1.2 (.9–1.6)	.146
40–50	469	142	1.8 (1.4–2.3)	<.001***	1.7 (1.3–2.3)	<.001***
50–60	331	104	1.9 (1.4–2.5)	<.001***	1.8 (1.3–2.4)	<.001***
60–80	107	31	1.7 (1.0–2.6)	.03*	1.7 (1.0–2.7)	.043*
Sex						
Male	496	138	1.0 (reference)	…	1.0 (reference)	…
Female	1865	455	0.8 (.7–1.1)	.118	0.9 (.7–1.2)	.6
Undetermined	17	0	…	…	…	…
Ethnicity						
White	1639	373	1.0 (reference)	…	1.0 (reference)	…
Any other	46	12	1.2 (.6–2.3)	.596	1.0 (.5–2.0)	.987
Arab	25	7	1.3 (.5–3.1)	.537	1.3 (.5–3.1)	.556
South Asian	333	88	1.4 (1.0–1.8)	.015*	1.2 (.9–1.6)	.23
Black	218	83	2.1 (1.6–2.8)	<.001***	1.7 (1.2–2.3)	.003**
Chinese	41	9	1.0 (.4–1.9)	.903	1.2 (.5–2.5)	.667
Mixed	76	21	1.3 (.8–2.1)	.325	1.2 (.7–2.1)	.427
Occupational characteristics						
Occupation						
AHPs	548	136	1.0 (reference)	…	1.0 (reference)	…
Cleaning/Catering/Porters	121	51	2.2 (1.5–3.3)	<.001***	1.4 (.8–2.2)	.196
Doctor	342	79	0.9 (.7–1.2)	.56	0.8 (.6–1.1)	.232
ICT	28	10	1.7 (.7–3.7)	.2	1.3 (.6–3.0)	.512
Manager	138	35	1.0 (.7–1.6)	.895	1.0 (.6–1.6)	.979
Nurse	859	205	1.0 (.7–1.2)	.684	1.1 (.8–1.4)	.592
Other	154	43	1.2 (.8–1.7)	.435	1.0 (.7–1.6)	.832
Scientist	188	34	0.7 (.4–1.0)	.06	0.7 (.4–1.0)	.064
Working from home						
No	1748	440	1.0 (reference)	…	1.0 (reference)	…
Yes	630	153	1.0 (.8–1.2)	.659	0.9 (.7–1.2)	.486
Regular income						
Yes	835	155	1.0 (reference)	…	…	…
No	100	33	2.2 (1.4–3.4)	.001**	…	…
Difficulty accessing sick leave^a^						
No	2134	497	1.0 (reference)	…	…	…
Yes	244	96	2.1 (1.6–2.8)	<.001***	1.8 (1.3–2.4)	<.001***
Environmental characteristics						
Public transport						
No	473	123	1.0 (reference)	…	…	…
Yes	637	128	0.7 (.5–1.0)	.02*	…	…
Known contact with COVID-19						
No	1496	328	1.0 (reference)	…	1.0 (reference)	…
Yes: household	263	75	1.4 (1.1–1.9)	.019*	1.6 (1.2–2.2)	.002**
Yes: other	60	22	2.1 (1.2–3.5)	.009**	1.9 (1.1–3.3)	.029*
Yes: patient	65	14	1.0 (.5–1.7)	.941	1.2 (.6–2.1)	.651
Yes: staff	402	125	1.6 (1.3–2.0)	<.001***	1.8 (1.4–2.4)	<.001***
Yes: travel	92	29	1.6 (1.0–2.6)	.034*	1.9 (1.2–3.0)	.008**
Crowding						
Multigenerational household						
No	2233	557	1.0 (reference)	…	1.0 (reference)	…
Yes	145	36	1.0 (.7–1.4)	.975	0.9 (.6–1.4)	.689
Household rooms						
No. of rooms	2378	…	1.0 (1.0–1.1)	.98	…	…
Household members						
No. of members	2378	…	1.1 (1.1–1.2)	<.001***	…	…
Children						
No. of children <5 y	864	…	1.0 (.7–1.2)	.774	…	…
Household index						
Household members/rooms	2378	…	1.5 (1.2–2.0)	<.001***	1.4 (1.1–1.9)	.006**
Socioeconomic characteristics						
Deprivation index quantile^b^						
4 (least deprived)	501	120	1.0 (reference)	…	1.0 (reference)	…
3	635	149	1.0 (.7–1.3)	.848	1.0 (.8–1.3)	.978
2	768	195	1.1 (.8–1.4)	.562	1.0 (.8–1.4)	.767
1 (most deprived)	474	129	1.2 (.9–1.6)	.243	1.1 (.8–1.4)	.76

Statistical significance of the logistic regression presented next to the *P* values.

Abbreviations: AHPs, allied health professionals; CI, confidence interval; COVID-19, coronavirus disease 2019; ICT, Information Computing Technology; OR, odds ratio.

^a^Do any working people in your household have difficulty accessing sick leave/pay?

^b^Index of multiple deprivation as reported by the United Kingdom Ministry of Housing, Communities and Local Government (www.gov.uk/government/statistics/english-indices-of-deprivation-2019).

^*^ *P <* .05.

^**^ *P <* .01.

^***^ *P <* .001.

### Occupational Risks of SARS-CoV-2 Infection or Exposure

Seropositivity varied by specialty; however, occupations with known exposure to aerosolizing procedures (anesthetics, pediatric intensive care unit [ICU], cardiac ICU) did not have increased risk of seropositivity relative to staff working on other inpatient or outpatient wards.

Univariate analysis identified cleaners, porters, and catering staff as having the highest risk of seropositivity at 42.1% (OR, 2.2 [95% CI, 1.5–3.3]; *P* < .001). The second highest occupational rate was in the Information Computing Technology (ICT) Department at 35.7%, though this was not statistically significant on univariate analysis (OR, 1.7 [95% CI, .7–3.7]; *P* = .512). One potential explanation for this finding is that ICT is a small department.

Overall, on multivariate analysis, no occupation was found to have a statistically significant risk of seropositivity (Figure 1). Clinical staff did not have higher rates of seropositivity than nonclinical staff, and working from home did not impact rates of seropositivity.

### Contact With COVID-19/Symptoms of COVID-19

In total, 34.4% of staff had a known contact with COVID-19 at the time of the survey. Compared to those that did not have a known contact with COVID-19, those with a known contact had an increased risk of seropositivity (30% compared to 21.9%), except if the known contact was a patient. Contact involving travel to Italy, China, Iran, or South Korea between the months of December 2019 and February 2020 (OR, 1.9 [95% CI, 1.2–3.0]; *P* = .008), other staff members (OR, 1.8 [95% CI, 1.4–2.4]; *P* < .001), household members (OR, 1.6 [95% CI, 1.2–2.2]; *P* = .002), and other SARS-CoV-2 contact (OR, 1.9 [95% CI, 1.1–3.3]; *P* = .029) all remained statistically significant on the multivariate analysis. Thirty-eight percent (347/911) of those staff who reported symptoms consistent with SARS-CoV-2 infection were seropositive, compared to 13% (365/2735) of asymptomatic staff.

### Socioeconomic Risks of SARS-CoV-2 Infection or Exposure

Staff that lived in households in which 1 or more household members did not have access to adequate sick leave had a statistically significant increased risk of COVID-19 (OR, 1.8 [95% CI, 1.3–2.4]; *P* < .001) (Figure 1). Staff who self-reported that their income was not always enough to cover basic needs of housing, transport, and food had higher rates of COVID-19: 33% compared with 15.7% in those who did not (OR, 2.2 [95% CI, 1.4–3.4]; *P* = .001). However, this was not included in the multivariate analysis as >30% of the entries contained missing values. Moreover, entries with missing data regarding income were not missing at random, with seropositive individuals characterized by a higher OR of income missing values and some ethnicities such as Black or Asian presenting lower ORs of income missing values when compared to White (Table 3). Areas of social deprivation based on postal codes were analyzed for COVID-19 risk. The quartiles of lowest to highest rates of deprivation did not show significant increased risk on multivariate analysis.

**Table 3.** Association Between Variables and Missing “Regular Income” Data

Variable	OR (95% CI)	*P* Value
Seropositivity	1.8 (1.5–2.2)	<.001***
Age, y		
17–30	1.0 (reference)	…
30–40	1.2 (.9–1.5)	.14
40–50	1.3 (1–1.7)	.057
50–60	1.6 (1.2–2.1)	.004**
60–80	1 (.6–1.6)	.94
Sex		
Male	1.0 (reference)	…
Female	1 (.8–1.2)	.9
Undetermined	1.5 (.5–4.9)	.45
Ethnicity		
White	1.0 (reference)	…
Any other	.5 (.3–.9)	.031
Arab	.5 (.2–1.2)	.12
South Asian	.7 (.5–.9)	.003**
Black	.6 (.4–.8)	<.001***
Chinese	1.2 (.6–2.5)	.55
Mixed	1 (.6–1.7)	.86
Occupation		
AHPs	1.0 (reference)	…
Cleaning/Catering/Porters	.3 (.2–.5)	<.001***
Doctor	1.1 (.8–1.5)	.65
ICT	1 (.4–2.4)	.99
Manager	1.1 (.7–1.7)	.62
Nurse	1.2 (.9–1.5)	.13
Other	0.7 (.5–1)	.03*
Scientist	1.7 (1.2–2.5)	.004**
Working from home		
No	1.0 (reference)	…
Yes	1.1 (.9–1.4)	.25
Difficulty accessing sick leave		
No	…	…
Yes	.6 (.5–.8)	.0013**
Known contact with COVID-19		
No	1.0 (reference)	…
Yes: household	1.5 (1.2–2.1)	.003**
Yes: other	1.1 (.6–2)	.7
Yes: patient	2 (1.1–3.6)	.021*
Yes: staff	1.5 (1.2–1.9)	.0012**
Yes: travel	1.3 (.8–2)	.29
Multigenerational household		
No	1.0 (reference)	…
Yes	.8 (.5–1.1)	.17
Household index		
Household members/rooms	.9 (.7–1.2)	.52
Deprivation index quartile		
4 (least deprived)	1.0 (reference)	…
3	1 (.8–1.3)	.82
2	1.1 (.9–1.4)	.45
1 (most deprived)	1.2 (.9–1.6)	.26

Statistical significance of the logistic regression is presented next to the *P* values.

Abbreviations: AHPs, allied health professionals; CI, confidence interval; COVID-19, coronavirus disease 2019; ICT, Information Computing Technology; OR, odds ratio.

^*^ *P* < .05.

^**^ *P* < .01.

^***^ *P* < 0.001.

### Environmental Risks of SARS-CoV-2 Infection or Exposure

Presence of children under the age of 5 in the household did not change seropositivity rates of COVID-19. Neither did households that had >2 generations of family members. An increasing number of household members marginally increased the risk of COVID-19 on univariate analysis (OR, 1.1 [95% CI, 1.1–1.2]; *P* < .001). More notable was that an increased ratio of household members relative to rooms in the house increased the risk of COVID-19, which remained significant on multivariate analysis (OR, 1.4 [95% CI, 1.1–1.9]; *P* = .006).

## DISCUSSION

This large prospective study of HCWs in a pediatric tertiary referral hospital demonstrated that lack of access to sick pay, relative household overcrowding, Black ethnicity, and increasing age were independently associated with SARS-CoV-2 seropositivity.

GOSH is a large tertiary pediatric hospital in central London. The hospital faced unprecedented demand as pediatric units across London were closed to increase adult bed capacity. However, thankfully, relative to adult centers, very few inpatients were severely infected with acute respiratory COVID-19 during the first wave of the pandemic. At GOSH, all inpatients were tested for COVID-19 on admission and were admitted to side rooms pending test results. Only 53 inpatients were diagnosed with COVID-19 during the period when this study was conducted. It is therefore unsurprising that contact with a patient with COVID-19 was not shown to be a risk factor for seropositivity in this study, whereas other types of contact with COVID-19 were. This is supported by findings by Goldblatt et al demonstrating low in-hospital transmission rates among HCWs in pediatric facilities in 8 European countries [24]. Moreover, clinical staff did not have higher rates of seropositivity than nonclinical staff. This suggests that seropositive staff members primarily acquired COVID-19 either from other staff members at work or outside work [25–27].

Structural inequalities related to socioeconomic status (SES) and ethnicity have been directly linked with COVID-19 [28–30]. HCWs from a lower socioeconomic background are more likely to live in overcrowded housing, which is a risk factor for respiratory illnesses [31]. Many studies use occupation as a surrogate for SES but have not investigated how income, job security, household environment, and living in an area of social deprivation may impact SARS-CoV-2 seropositivity [5, 6, 32]. Studies of HCWs in other UK hospitals found that HCWs from minority ethnic groups had a significantly increased risk of seropositivity (OR, 1.92 [95% CI, 1.14–3.23]; *P* = .01) [7].

Staff in nonclinical roles such as cleaners, porters, and catering staff had the highest risk of acquiring SARS-CoV-2 prior to consideration of confounders in a multivariate analysis. This observed higher risk among nonclinical support staff compared to clinical staff has also been reported by other studies from the UK, Norway, and the United States [12, 13, 33]. The fact that occupation did not remain a significant risk factor after controlling for confounders suggests that the postulated association between occupational risk and SARS-CoV-2 seropositivity is actually due to underlying demographic and socioeconomic factors.

Lack of access to sick pay was independently associated with higher rates of SARS-CoV-2 seropositivity. In the UK, 27% of NHS Estates and Facilities workers (which includes cleaners, porters, catering, security, engineering, capital delivery, and maintenance staff) are outsourced to service delivery partners, 7% are employed by NHS wholly owned subsidiaries, and 66% are directly employed by the NHS [34]. The ONS reports that as of April–June 2022, 20.2% of workers in the field of health and social care are on zero-hours contracts, and a Freedom of Information request submitted by the *Financial Times* found that in 2013, NHS hospitals used almost 100 000 zero-hours contracts [35, 36]. While zero-hours contracts are legal in the UK [37], lower-paid staff members who cannot afford to lose income are more likely to work when they are unwell or have had a COVID-19 contact [38]. This economic vulnerability may make self-isolation challenging and could contribute to the spread of COVID-19 and other infectious diseases among nonclinical healthcare staff. The US Centers for Disease Control and Prevention has highlighted the importance of avoiding incentives that encourage people to come to work when symptomatic [39].

Overcrowded housing is a recognized risk factor for respiratory and other infectious diseases, and overcrowding affects 3% of households in England, with ethnic minorities disproportionately affected [40, 41]. It is therefore unsurprising that there was an increased risk of seropositivity with a higher ratio of household members to rooms (OR, 1.4 [95% CI, 1.1–1.9]; *P* = .006). Overcrowded housing is also considered a measure of poverty and further highlights the impact that SES has on COVID-19 infection among HCWs [42].

The role that ethnicity plays in SARS-CoV-2 seropositivity has been discussed by many preceding studies [43–45]. We found a significant association between seropositivity and both South Asian and Black ethnicity on the univariate analysis. This correlates with findings from other studies in the UK [6, 28]. Studies in the United States have shown that Black and South Asian workers are more likely to be employed in healthcare, social assistance, and other essential industries [46, 47]. ONS data have shown that while minority ethnic groups have higher rates of death from COVID-19, much of this difference is attributable to socioeconomic factors, living conditions, and occupational exposure [48, 49]. In the UK, poverty rates are the highest in the Bangladeshi (65%) community, followed by Pakistani (55%), Black African (45%), Black Caribbean (30%), Indian (25%), and White British (20%) [31]. On multivariate analysis, only Black ethnicity remained significant, whereas South Asian ethnicity did not. There may be other unmeasured confounding factors that contribute to the Black ethnic risk of SARS-CoV-2 infection that we have not identified in our analysis.

This study benefited from a large, diverse, engaged cohort of recruited HCWs. Other strengths included the collection of data on a wide range of demographic, occupational, and socioeconomic factors, as well as data on exposure to COVID-19. This allowed a detailed consideration of the influence of socioeconomic and environmental variables, which are often overlooked. Our study design ensured that our cohort was truly representative of the entirety of HCWs in the hospital by actively recruiting cleaners, porters, and catering staff, who were harder to reach due to lack of access to NHS Trust emails (these staff members at the time were employed by external organizations and so may not have had NHS email accounts). This enabled us to achieve similar recruitment levels for all staff groups. Additionally, our data were gathered over a relatively short time period (April–September 2020), and we used the MSD assay to test antibodies against SARS-CoV-2 [19, 50], meaning that we would not expect antibodies to have waned during this time period. Consequently, seropositivity rates are more likely to be a true reflection of exposure to COVID-19.

There are, however, some important limitations. Data including some socioeconomic factors such as difficulty accessing sick leave were self-reported and could be subject to reporting bias. These data could be useful to elucidate whether this behavior was driven primarily by a lack of contractual sick leave, or by perceptions about the effects of taking sick leave on their employment—which may be driven by wider socioeconomic and workplace factors. Staff on lower incomes may feel unable to isolate even if they do have access to statutory sick leave, influenced by their overall economic precarity, concern about repercussions, or a sense of duty to their work. There was also a small proportion of staff who declined to participate in the study (36 participants or 0.99% of those recruited), and the lack of their demographic and occupational data makes it difficult to determine the extent that this may have influenced transmission.

The COVID-19 pandemic highlighted the effect of structural and ethnic inequality on communicable disease in the UK [48]. This is corroborated in our data demonstrating an increased risk of SARS-CoV-2 seropositivity in staff reporting difficulty accessing sick leave, those living in overcrowded housing, and those of Black ethnicity. These data emphasize the importance of taking economic, ethnic, and social factors into account when forming public health policy, and underscore the impacts of social determinants of health in the UK. Since the pandemic, cleaning staff at GOSH have been brought in house, under full hospital employment, and several NHS trusts have done the same [51]. This should be considered around the country to ensure that all staff have equitable access to information, participation in the life of the hospital, and a safe place to work.

## Supplementary Data

Supplementary materials are available at *Clinical Infectious Diseases* online. Consisting of data provided by the authors to benefit the reader, the posted materials are not copyedited and are the sole responsibility of the authors, so questions or comments should be addressed to the corresponding author.

## Supplementary Material

ciad522_Supplementary_Data

## Notes

***Acknowledgments*.** The authors dedicate this article to the staff members at Great Ormond Street Hospital (GOSH) who died of coronavirus disease 2019 (COVID-19) during the first wave of the pandemic. The authors also thank all of the staff at GOSH who took part in the study. In addition, the authors are grateful for all the hard work undertaken by the Great Ormond Street laboratory staff and the staff in the immunology laboratories, both in the Camelia Botnar Laboratory and the Great Ormond Street Institute of Child Health, who ensured that the polymerase chain reaction tests and serological assays were completed in a timely manner. Finally, the authors acknowledge the support of the GOSH Research and Development, Governance, Finance, Management, Estates, Operations, and Communications departments.

***Co-STARs Study Team.*** Dorcas Mirambe-Korsah, Fernanda Fenn Torrente, Jakub Wyszynski, Victoria Gander, Amy Leonard, Louise Myers, Aimee Vallot, Camille Paillas, Rose Fitzgerald, Adam Twigg, Rabia Manaf, Lois Gibbons, Hollie Powell, Richard Nar-Dorh, Ally Gray, Elias Fernandez, Aline Minja, Emily Beech, Waffa Girshab, Pei Shi Chia, Kate Webb, Malti Nakrani, Kim Gardiner, Valerija Karaluka, Karen Ryan, Dorothy Lee, Katie Groves, Hamad Khan, Shamime Nsubuga, Olivia Rosie-Wilkinson, Julia Spires, Nuria Sanchez-Clemente, Sapriya Kaur, Natasha Carroll, Jemma Efford, Gabriel Bredin, Celma Marisa Dos Santos Domingues, Sophie Foxall, Helen Ashton, Abbey Afzal, Sally Mainland, Kate Crumpler, Lucinda Dawson, Claire Smith, Maria Tabbu, Laura Chiverton, Jade Sugars, Jordan Mooney, Dorothy Chikusu, Fariba Tahami, Baratth Samy, Shomona Begum, Dhimple Patel, Philippa Wiltshire, Annie Susay, Anna Ryan, Luke Lancaster, Kavita Thind, Kate Speller, Rachel Sterling, Connor Tugulu, Sandhya Ghurburrun, Steffi Gray, Joy Mugas, Moe Kishma, Kathleen Akpokomua, Sophie White, Eleana Pieri, Sabina Shamsad, Demi Alexandrou, Odera Aguele, Katherine Miles, Anamika Jain, Subishma Gautam, Oliver Simms, Rachel Goff, Zarif Shams, Tinya Chirinda, Aaliya Nur, and Tarekur Rahman.

***Disclaimer.*** The funders had no role in the study design, data collection and analysis, decision to publish, or preparation of the manuscript.

***Financial support*.** L. G. was supported by the Wellcome Trust (award numbers 201470/Z/16/Z and 226007/Z/22/Z); the National Institute of Allergy and Infectious Diseases of the National Institutes of Health (award number 1R01AI146338); the GOSH Charity (award number VC0921); and the GOSH/Institute of Child Health Biomedical Research Centre (www.nihr.ac.uk). A. S. was supported by the Wellcome Trust (award number 220565/Z/20/Z).
