# Supplementary material for: Socioeconomic and Demographic Risk Factors for SARS-CoV-2 Seropositivity Among Healthcare Workers in a UK Hospital: A Prospective Cohort Study
Source: Clin Infect Dis. 2023 Aug 30;78(3):594–602. doi: 10.1093/cid/ciad522 (PMC10954340; doi:10.1093/cid/ciad522)
Supplement: ciad522_Supplementary_Data [file ciad522_supplementary_data.docx]

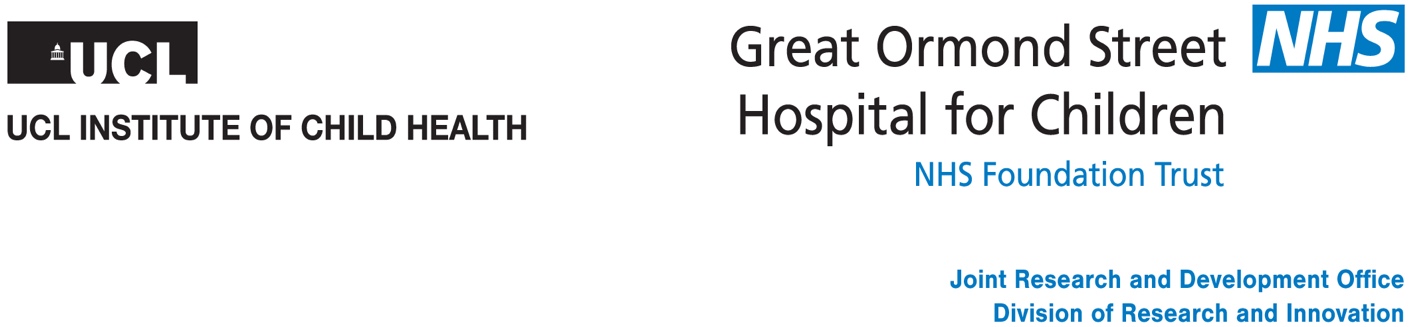


**Study Title: COVID-19 Staff Testing of Antibody Responses Study (Co-STARS)**

**Protocol Number: 20CB17**

**IRAS ID: 282713**

**Protocol Version: *(Version 2 )***

**Short title or acronym: The Co-STARS Study**

| **Chief Investigator:** | Dr. Louis Grandjean, [louis.grandjean@gosh.nhs.uk](mailto:louis.grandjean@gosh.nhs.uk), Department of Infectious Diseases, |
| --- | --- |
| **Investigators:** | Professor David Goldblatt, [d.goldblatt@ucl.ac.uk](mailto:d.goldblatt@ucl.ac.uk)  Professor Judith Breuer, [j.breuer@ucl.ac.uk](mailto:j.breuer@ucl.ac.uk)  Dr. Claire Smith, [c.m.smith@ucl.ac.uk](mailto:c.m.smith@ucl.ac.uk)  Dr. Kimberly Gilmour, kimberly.gilmour@gosh.nhs.uk  Dr. James Hatcher, [james.hatcher@gosh.nhs.uk](mailto:james.hatcher@gosh.nhs.uk)  Dr. Tanya Lam, [tanya.lam@gosh.nhs.uk](mailto:tanya.lam@gosh.nhs.uk)  Dr. Anja Saso, anja.saso@nhs.net |
|  |  |
| **Funder:** | Currently Unfunded |

Conflict of Interest: None for any investigators

**Sponsor**

Great Ormond Street Hospital For Children NHS Foundation Trust

Joint R&D Office GOSH/ICH based at UCL Institute of Child Health

30 Guilford Street

London

United Kingdom

WC1N 1EH

vincent.grek@o4cp.comEmail: research.governance@gosh.nhs.uk

**Signatures**

The Chief Investigator, Principal Investigators and Sponsor have discussed this protocol. All have agreed to perform the investigation as written and to abide by this protocol except in case of medical emergency or where departures from it are mutually agreed in writing.

___________________________________
Sponsor Signature

Date:

**Chief Investigator**


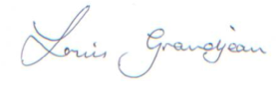


___________________________________

Signature

Date: 20^th^ May 2020

**Participating Sites and Local Principal Investigators (PI)**

Primary Site: Great Ormond Street Hospital for Children NHS Foundation Trust

A further list of sites doing concurrent studies who will compare data with GOSH are available in Appendix D. These sites will be responsible for their own protocols, HRA and ethics applications.

#

# Amendment History

| **Amendment No.** | **Protocol Version No.** | **Date issued** | **Author(s) of changes** | **Details of Changes made** |
| --- | --- | --- | --- | --- |
| Substantial Amendment 01 | Version 2 | 1st June 2020 | Louis Grandjean | Addition of PI Dr Anya Saso  Increasing Predicted Sample Size to 7000  Inviting staff who have been tested already in the staff testing program to join the study.  Results return by email, text and phone. Development of questionnaire that can be accessed by mobile devices.  For seropositive group, an optional extra blood sample for T-cell assays and optional IgA on salivary samples. More detail added to the neutralization assays that will be undertaken.  Testing of sera at Professor Golblatt’s UCL/ICH laboratory. The ability to run other serological tests in parallel. Extra sites that will collaborate with shared anonymised data.  Informing participants of other COVID-19 studies being undertaken at GOSH and affiliated sites that they are eligible to participate in. These include but are not limited to: neuro-radiological findings in COVID-19 disease, PPE mask testing study, Genetically modified T-cell therapy study, IgA diagnostics study, Genomics of severe and mild disease.  Additional questions on prior testing, risk factors, severity of disease, complications and vaccinations added to questionnaire. |

# Abbreviations

| CI | Chief Investigator |
| --- | --- |
| CRF | Case Report Form |
| GCP | Good Clinical Practice |
| GOSH | Great Ormond Street Hospital |
| ICF | Informed Consent Form |
| ICH | Institute of Child Health |
| NHS | National Health Service |
| PI | Principal Investigator |
| PIL | Participant/ Patient Information Leaflet |
| PPE | Personal Protective Equipment |
| R&D | NHS Trust R&D Department |
| REC | Research Ethics Committee |
| SOP | Standard Operating Procedure |
| UCL | University College London |
| UCLH | University College London Hospital |

# Study Synopsis

| Title |  | **COVID-19 Staff Testing of Antibody Responses Study (Co-STARS)** |
| --- | --- | --- |
| Sponsor name |  | Great Ormond Street Hospital for Children NHS Foundation Trust |
| Primary objective |  | To determine the kinetics (rate of change) of SARS-CoV-2 specific antibody titres in proven cases of SARS-CoV-2 over the 6 month period following infection. |
| Secondary objective (s) |  | To determine the proportion of completely asymptomatic healthcare workers who have evidence of SARS-CoV-2 antibodies in their serum indicative of past infection.  To determine the attack rate of SARS-CoV-2 in healthcare workers who have antibodies versus those who do not have antibodies.  To determine the immune correlates of protection (antibody titres sufficient for protection) against future exposure to SARS-CoV-2.  To investigate the roles of T-cell function and IgA in those with seropositivity to SARS-CoV-2.  To examine key risk factors influencing susceptibility to SARS-CoV-2. These include but are not limited to social determinants of health, such as housing, work, lifestyle and transport in the urban setting.  To ascertain clinical manifestations of COVID-19 disease and long term consequences of disease.  To pool international data in standardised form across hospital sites for longitudinal comparative review of risk factors and health service impact.  To assess the impact of COVID-19 on health worker staffing in hospitals and inform strategies for future planning.  To review antibody responses to vaccination against SARS-CoV-2 following it’s availability and analyse subsequent infection rates. |
| Study Design |  | Prospective Cohort Study |
| Study Endpoints |  | 6 year 3 month follow up study |
| Sample Size |  | This study will recruit dependent on logistics, funding and demand. We have based our power calculations on what is currently possible with a starting cohort of N=~7000 healthcare workers ~2100 seropositive health care staff and ~4900 seronegative healthcare staff as per Study Flow diagram 1. |
| Summary of eligibility criteria |  | All healthcare workers >18 years of age at GOSH (excluding those on immunosuppressive medication, an immunodeficient condition and those who have already received convalescent sera) |
| Intervention |  | Repeated cross sectional surveys and blood sampling to measure antibody titres to the SARS-CoV-2 virus in healthcare workers over time |
| Procedures: Screening & enrolment |  | Staff will be sent emails with study information and eligibility criteria. Those opting for participation will be contacted for consent (via a face to face meeting, or if preferred a telephone consent or zoom meeting to avoid unnecessary mixing). After providing informed consent, participants undertake an online questionnaire, then attend an appointment for blood testing. |
| Baseline |  | May 2020 |
| Treatment period |  | 6 years |
| End of Study |  | May 2026 |

#

# Table of Contents

[Sponsor 1](#_Toc39696132)

[Participating Sites and Local Principal Investigators (PI) 3](#_Toc39696133)

[1 Amendment History 4](#_Toc39696134)

[2 Abbreviations 5](#_Toc39696135)

[3 Study Synopsis 6](#_Toc39696136)

[4 Table of Contents 8](#_Toc39696137)

[5 Introduction 9](#_Toc39696138)

[5.1 Background and Rationale 9](#_Toc39696139)

[6 Objective and purpose 13](#_Toc39696140)

[7 Study Design 13](#_Toc39696141)

[7.1 Description of study design 13](#_Toc39696142)

[8 Population 15](#_Toc39696143)

[8.1 Inclusion Criteria (See Also Study Flow Diagram 1 page 18) 16](#_Toc39696144)

[8.2 Exclusion Criteria 17](#_Toc39696145)

**8.3 Study Flow Diagram……………………………………………………………………………………….. 18**

[9 Study Procedures 19](#_Toc39696146)

[9.1 Recruitment 19](#_Toc39696147)

[9.2 Informed Consent 20](#_Toc39696148)

[9.3 Screening and Eligibility Assessment 21](#_Toc39696149)

[9.4 Baseline Assessments 22](#_Toc39696150)

[9.5 Subsequent Visits 22](#_Toc39696151)

[9.6 Study Duration 23](#_Toc39696152)

[9.7 Discontinuation/Withdrawal of Participants from Study 23](#_Toc39696153)

[9.8 Definition of End of Study 23](#_Toc39696154)

[10 Intervention 23](#_Toc39696155)

[11 Subject Withdrawal Criteria 24](#_Toc39696156)

[12 Statistics 24](#_Toc39696157)

[12.1 Statistical methods to be employed (plan of analysis) 22](#_Toc39696158)

[13 Data Management 27](#_Toc39696159)

[13.1 Source Documents 27](#_Toc39696160)

[13.2 Direct Access to source data / documents 28](#_Toc39696161)

[13.3 Data Recording and Record Keeping 28](#_Toc39696162)

**13.4 Archiving……………………………………….………………………………………………………………..29**

[14 Patient Confidentiality & Data Protection 29](#_Toc39696163)

[15 Sample Collection, Storage, Transfer and Analysis 30](#_Toc39696164)

[16 Financial Information and Insurance 32](#_Toc39696165)

[17 Publications Policy 32](#_Toc39696166)

[18 References 32](#_Toc39696167)

[19 Appendix A: Study Flow Charts 36](#_Toc39696170)

20 Appendix B: Schedule of Procedures 38

**21 Appendix C: Questionnaire……………………………………………………………….…………………39**

**22 Appendix D: Sites of collaboration conducting similar studies…….……………………….40**

#

## Introduction

## Background and Rationale

Since appearing as a cluster of patients with ARDS in the December of 2019 in Wuhan, China, COVID-19 caused by SARS-CoV-2 has rapidly spread worldwide, with pandemic status declared in March 2020.^1^ Currently there have been over 1.6million cases, with over 100, 000 deaths.^2^

SARS-CoV-2 is a β-coronavirus, an enveloped non-segmented positive-sense RNA virus.^3^

Sequencing has demonstrated that SARS-CoV2 is 79% identical to SARS-CoV, which caused the SARS (Severe Acute Respiratory Syndrome) epidemic in 2003, 50% with the MERS (Middle Eastern Respiratory Syndrome) coronavirus and 96% identical to a bat coronavirus.^4,5^ Analysis of 104 strains of samples taken between December and February demonstrated 99.9% homology, with little mutation, providing some hope for eventual vaccine therapy.^6,^ ^5^

The virus was first recognised in Wuhan, China. Though many of the initial cases were linked to a seafood market, a significant proportion of early cases did not have any association with the market, suggestive that human to human spread occurred in the month prior.^1^

In contrast to the related coronavirus respiratory diseases MERS and SARS, which were more frequently associated with nosocomial infections, COVID-19 spreads more avidly, via close contacts, with a household secondary attack rate of 3-5%.^7,6^ Early calculations of the reproductive number (R_0_) were estimated at 2.3-3.5.^8^ The viral load profile is similar to influenza, peaking early together with symptom onset, which could account for the higher transmissibility compared with SARS and MERS.^9^ The virus is spread through respiratory droplet and fomite spread in close proximity contact and via aerosolised procedures, a particular risk to health care workers. The virus has also been isolated in faecal samples, however no known cases of faecal-oral spread have been noted.^10,11^ Similarly to SARS-CoV, SARS-CoV-2 gains entry into respiratory epithelial cells via attachment to the ACE2 receptor.^4,12^ The clinical presentation and pathology resembles SARS and MERS, though with less upper respiratory and gastrointestinal symptoms.^13^ The estimated incubation period has a mean of 5 days, with the majority of cases developing symptoms by 14 days.^14^ However one study found 15.6% of patients had not developed symptoms by day 14 following exposure.^15^

Typical symptoms include fever, dry cough, dyspnoea and lethargy. Additional noted symptoms are headache, anosmia, haemoptysis.^16,17^ Whereas in SARS and MERS fevers occurs in 98-99% of cases, in COVID-19 a majority (55%) of patients did not have fever at presentation at hospital and 12% did not subsequently develop it.^7^ Fifteen percent of cases progress to severe disease, comprising respiratory compromise and ARDS, with the association phenomenon of silent hypoxemia, particularly in older patients.^7^ Time course to respiratory failure is usually >7 days, longer than in MERS and the mean age of 50 years is older than that of both SARS and MERS.^18^ Biopsy has revealed alveolar damage, with cellular fibromyxoid exudates and hyaline membrane formation. Histological features are similar to those seen in SARS and MERS.^19^

Data from 21 hospitals in China reveal that of those cases that died, 75% were male, 96% over 50 years, 70% had co-morbidities, most commonly hypertension, diabetes and ischaemic heart disease.^20^ In America 90% of hospitalized patients had an underlying condition, most commonly obesity, hypertension, chronic lung disease, diabetes mellitus, and cardiovascular disease.^21^

Asymptomatic infection rates remain an important question that will impact public policy and the need for ongoing levels of isolation. Rates calculated on the affected Diamond Princess cruise ship were 17.9%.^22^ Since then discussions by epidemiologists have suggested a significantly higher asymptomatic carrier rate, with recent new cases in China being up to 80% asymptomatic.^23^

Diagnostic testing for SARS-CoV-2 has been based on Nasopharyngeal swab RT-PCR. Target selection for RT-PCR tests vary between countries and there isn’t consensus on their accuracy.^24^ Accuracy is affected by sampling location and method, quality of RNA extraction and assay and training of operators. Reporting on the sensitivity of RT-PCR described rates below 50%, emphasising the need to consider the clinical picture and background likely prevalence in formulating diagnosis.^25,26^ Multiple studies have published on the sensitivity of serological testing purporting to a sensitivity above 80%, significantly higher than PCR testing of oral swabs or blood samples.^27,28,29^

- Lui studied 133 moderate to critical patients in Wuhan and found IgM had a higher sensitivity than RT-PCR (79% vs 68%). However, both tests reported false positive and false negative results.^30^
- Zhang’s study of 222 patients in Wuhan during the convalescent stage (within 35 days) found 98.6% had IgG detected and 82% had IgM detected. IgG was first detected on day 4 of illness and peaked in the 4^th^ week, IgM was first detected on day 3 of illness and peaked in the 2^nd^ week.^31^
- Lou et al tested eighty patients (confirmed on PCR with deep sputum samples) using ELISA, lateral flow assays and CMIA (chemiluminescence microparticle immunoassays). ELISA performed the best of all three tests but the difference was not found to be significant. IgM and IgG seroconversion occurred at 10- and 12-days post symptom onset respectively. Antibody levels increased rapidly after day 6 post onset and by day 21 IgM seroconversion was 100% and by day 29 IgG was 97.1% (93.3% at 21days). Decline of viral load co-occurred with the rising antibody levels. At day 14 post exposure 45.5% had seroconverted. Keeping note of isolation duration times, 15.6% did not present with symptoms prior to 14 days post exposure.^15^
- To’s study of 23 patients, found at 14 days seropositivity against internal nucleoprotein (NP) was 94% for IgG and 88% for IgM. Against nucleo-capsid protein receptor binding domain (RBD), it was 100% for IgG and 94% for IgM. More patients seroconverted to IgG before IgM for both types, but this may be due to reduced sensitivity of the IgM EIA. Viral load was inversely related to antibody response. However in Zhao’s study three critical patients did not have a decline in viral load associated with increasing antibody titres.^32^ Most patients developed antibodies around day ten, one patient with severe disease developed antibodies at day 6. No genomic mutations were detected on serial sampling from four patients.^9^
- Zhao studied 173 patients and found whilst in the first 7 days following onset, PCR was more sensitive than serological tests (66.7% vs 38%), By day 12 seroconversion rates were 90% whilst PCR detection was 54% and by days 15-30 PCR detection was 45.5%. Of the patients that did not seroconvert – all samples were taken before day 13 of symptom onset.^32^ Thus, serology is especially important for patients that present late, with viral loads below the detection limit of RT-PCR.^9^

Serological testing thus supports clinical management and will provide a key measure of the number asymptomatic cases, helping to understand the epidemiology of the virus and the true mortality rate among as a proportion of all infections. Serological testing will also assist in understanding the role of children in transmission, who comprise a low attack rate of 2.4% of cases.^6^. Furthermore, knowledge of what antibody titres are protective will enable us to track the protective duration of vaccination when this is made widely available.^33^

The sensitivity and specificity of serological testing for SARS-CoV-2 can be affected by the mode of testing. Rapid antigen lateral flow assays have the benefit of fast time to results but are likely to suffer from poor sensitivity, which has been the experience with influenza tests. Sensitivity has been as low as 60% for some lateral flow assays with the usual pre-FDA-approval test performance metrics being bypassed to expedite the availability of testing.^34^ IgM responses are often non-specific, and limit the applicability of this test for diagnosis and active management. IgG is more specific, but can take weeks to appear.^24^ In Zhao’s study median IgM was 12 days after onset and IgG was 14 days after onset.^32^

Cross reactivity with other coronaviruses must also be excluded. The abundantly expressed internal NP has 90% amino acid homology to SARS-CoV and may have potential cross-antigenicity, whereas the surface nucleo-capsid RBD is specific for SARS-CoV-2.^9^ The nucleo-capsid protein is the sole protein on the surface of the virus that is responsible for entry into human cells. It is highly conserved and abundant, thus easy to detect.^12^ This is supported by the findings of To’s study above.

In the preceding coronavirus respiratory illness epidemics, long term antibody testing demonstrated presence of neutralising antibodies for at least two years in patients following SARS, with a rapid decline in the 3^rd^ and 4^th^ years.^35,36,37^ A group of 34 health care workers were followed for SARS-CoV IgG and some had persistence up to 12 years after infection.^38^ Singapore recently reported that a patient continued to have SARS antibodies 17 years after infection.^39^ In MERS, antibodies persisted for at least 34 months in 86% (6 of 7) patients.^40^

In a study of 301 SARS patients, seroconversion was detected from day 4 of illness. Early seroconversion (before 16 days) occurred more frequently among patients who required ICU admission and higher IgG levels were associated with patients who required oxygen therapy.^41^ In another study, SARS patients who died had an early IgG response that wasn’t sustained, whereas, in patients that recovered, IgG levels were slower to peak (average of 20 days) and were sustained for longer.^42^ Zhang has also suggested that higher IgG predicts disease severity in COVID-19.^31^

To date, no studies have been published regarding whether infection with SARS-CoV-2, protects against re-infection. A study of four rhesus monkeys infected with SARS-CoV-2, found that reinfection challenge of two monkeys at day 28, did not produce detectable virus excretion from nasopharyngeal and anal swabs (followed for 14 days in one monkey), or histopathological changes in tissues (on day 5 when necrotised). All monkeys had neutralising antibodies and none of the two re-exposed monkeys exhibited antibody-dependent enhancement (ADE).^43^ However this study is limited by the low number of animals used and the fact that one of two animals re-exposed was euthanised 5 days after exposure not allowing enough time for potential clinical disease to arise.

The relationship between antibody levels, viral loads and asymptomatic shedding remains a key focus of research. This knowledge will enable an improved understanding of transmissibility and pathogenesis.^44^. After discharge from hospital some patients remain or return to viral positivity and even relapse. This indicates that a virus -eliminating immune response may be difficult to induce in some patients and vaccines may not work in these individuals.^45^ Multiple sources suggest that recent sporadic reports of COVID-19 re-infection cases are more likely due to limitations of PCR testing techniques. In To’s study, several patients tested positive after serial negative results during the same admission.^43,46,9^*^,^*^39^*^,^*^12^

The World Health Organization’s (WHO) outline of key knowledge gaps includes immunity and immune diagnostics. These highlighted the questions of strength and duration of immunity, reflection of immunity by antibody tests, sero-specificity and co stimulation in serological diagnostics.^47^

We propose a longitudinal cohort study of health care staff to address the key questions of immunity to SARS-CoV-2 infection.

1. For how long and at what titres are antibodies detectable following symptomatic, PCR positive healthcare workers with confirmed infection?
2. What is the proportion of completely asymptomatic healthcare workers with serological evidence of infection?
3. What is the attack rate and incidence of SARS-CoV-2 infection among seronegative and seropositive healthcare workers?
4. What antibody titres are protective of reinfection?

Knowledge of the serological status of healthcare staff will help enumerate the true number of infections in this group, revealing the percentage of asymptomatic infections, the rates of seroconversion, antibody titres and duration of seroprevalence. Notably none of the studies mentioned above followed asymptomatic, non-hospitalised patients, so very little is known about this group.

We have acquired and performance tested a semi-quantifiable ELISA assay (*EDI^TM^*­ diagnostics, California) with appropriate positive and negative controls. Of 20 staff members who were proven PCR positive on nasal and pharyngeal swabs, 19 were also serologically positive by this ELISA after 14 days following the onset of symptoms whilst 15/15 negative controls – including adult sera from pre-2019, sera from known seasonal coronavirus with types OC43, HUK1, 229E and NL63 and serum from patients with hyper-immune phenotypes – all screened negative for coronavirus nucleo-capsid antibodies.

Close follow up of repeatedly exposed healthcare workers to identify potential infection/re-infection with SARS-CoV-2 will also indicate what level of protection is conferred by seropositivity. This information will eventually help us protect staff during further waves of the pandemic.

# Objective and purpose

| **Objectives** | **Outcome Measures/Endpoints** |
| --- | --- |
| **Primary Objective** | To determine the kinetics (rate of change) of SARS-CoV-2 specific protein antibody titres in proven cases of SARS-CoV-2 over the 6 month period following infection. |
| **Secondary Objectives** | 1. To determine the proportion of completely asymptomatic healthcare workers who have evidence of SARS-CoV-2 antibodies in their serum indicative of past infection 2. To determine the attack rate of SARS-CoV-2 in healthcare workers who have antibodies versus those who do not have antibodies 3. To determine the immune correlates of protection (antibody titres sufficient for protection) against future exposure to SARS-CoV-2 4. To investigate the roles of T-cell function and IgA in those with seropositivity to SARS-CoV-2. 5. To examine key risk factors influencing susceptibility to SARS-CoV-2. These include social determinants of health, such as housing, work and transport in the urban setting. 6. To ascertain clinical manifestations of COVID-19 disease and long term consequences of disease. 7. To pool international data in standardized form across hospital sites for longitudinal comparative review of risk factors and health service impact. 8. To assess the impact of COVID-19 on health worker staffing in hospitals and inform strategies for future planning. 9. To review antibody responses to vaccination against SARS-CoV-2 following it’s availability and analyse subsequent infection rates. |

# Study Design

## Description of study design

This single centre prospective cohort study will recruit at ~7000 members of Great Ormond Street Hospital (GOSH) staff and follow them up over 6 years 3 months. The number of follow-up re-tests will depend on logistics, demand and funding.

Hospitals nationally and internationally have shown interested in the protocol and if they commence concurrent studies, anonymised results can be compared. Anonymised samples and data may be shared between institutions. No identifiable data will leave GOSH Trust. These collaborating sites are listed in the table 3 and include Imperial Health, Alderhey Hospital , Chalfont Centre (UCLH) and European sites who may send samples to Goldblatt laboratory in UCL/ICH for cross-sectional analysis of prevalence. These include children’s hospitals in Lithuania, Latvia, Estonia, Romania, Finland, Greece, Austria, Iceland, Belgium, Spain, South Africa and Ireland. Once local ethical approval has been approved in other settings, we will facilitate the transfer and processing of samples at the infection and immunity laboratories of UCL ICH under a Materials Transfer Agreement between the organizations in order to transfer the sera between countries. Whilst Co-STARS is a single site study, in order to maximize power for detection of recurrent infections we will completely align our study with the SIREN project led by Susan Hopkins at Imperial College. Equally the SIREN study will adapt their protocol to mirror aspects of the Co-STARS study. Large institutional collaboration of this kind is necessary to diminish the time required to estimate the incidence of secondary disease versus primary infection.

For the purposes of statistical analysis we have based our study size calculations on this starting total cohort size of ~7000 participants with ~2100 seropositive participants. Any increase in sample size will improve study power and the ability to detect smaller effect sizes.

Within this initial planned cohort (**Study Flow Diagram 1, page 18**), a subset of ~400 staff members with confirmed symptomatic, PCR positive, antibody positive or equivocal, SARS-CoV-2 disease (and an additional 2100 staff sero-positive or equivocal staff members who were either PCR negative or asymptomatic) will be followed with intensive 1 to 2 monthly blood sampling to determine antibody titres to the SARS-CoV-2 specific protein using the *EDI^TM^* ELISA assay. Participants who are seropositive or equivocal will also have the option of providing an additional one off 20ml sample for T-cell assay analysis and a salivary sample for IgA analysis.

All 7000 recruited healthcare workers (2100 SARS-CoV-2 sero-positives and N~4900 seronegatives) will have blood taken for serological testing at baseline then at 6-monthly intervals for a total of 6 years. In addition to repeated serological testing, participants will continue as all healthcare workers at GOSH to have access to the staff testing program. This program tests symptomatic staff members by nasal swab PCR testing for SARS-CoV-2. Staff who have been tested for SARS-CoV-2 through the staff testing program will be consented for the PCR and serology results to be used in the study. All participants will also be asked to complete a detailed health and demographic questionnaire prior to each appointment, electronically. The technology design of the questionnaire will optimise participation. If key questions are missed, participants will be contacted by phone, text or email to optimise data collection and statistical analysis. As key factors impacting COVID-19 evolve, questions on review questionnaires will address these.

The data will enable us to determine the attack rate (percentage of healthcare workers with PCR proven infections/re-infections) and incidence (the rate of occurrence of disease) of SARS-CoV-2 infection amongst healthcare workers with and without antibodies. The comparison of the attack rate will indicate the protection antibodies provide. Further, the stratification of results by antibody titres, will provide information on whether a specific level of antibody confers protection.

Careful consideration will be given to the way in which recruited staff are provided with results. Specifically, it will be explained that at this stage, the test is research-based. Therefore it is not certain what the significance of positive serology means with respect to future risk of re-infection. It will be emphasised that a positive serological test does not abrogate the need to wear personal protective equipment, self-isolate or test for SARS-CoV-2 as per government guidelines.

# Population

The population will comprise frontline healthcare workers selected to be representative of all departments at Great Ormond Street Hospital. We will ensure that that age ranges and sex are representative of the trust population age distribution. The age distribution of the participants will be checked during recruitment against the known age distribution of the hospital to ensure a representative sample. If significant differences among ages between the study population and the hospital population age range emerge, the study will actively recruit age ranges underrepresented. If participants leave the GOSH NHS trust during the study they will remain eligible to continue in the study. Their contact details will be updated on follow-up questionnaires.

## Inclusion Criteria (See Also Study Flow Diagram, page 18 )

***SARS-CoV-2 Seropositive Cases N ~ 2100***

- Healthcare worker at GOSH (other UK and European Hospital Healthcare workers at their corresponding sites)
- ≥18 years of age
- ALL have confirmed detectable antibodies to SARS-CoV-2 infection on baseline screen

Then 1 of:

**Core Group of PCR Confirmed Cases**

## Symptoms consistent with SARS-CoV-2 infection and SARS-CoV-2 PCR Positive N ~ 400

OR

**Other SARS-CoV-2 Sero-positives N ~ 1700**

B) Symptoms consistent with SARS-CoV-2 infection and SARS-CoV-2 PCR Negative cases

OR

C) No symptoms consistent with SARS-CoV-2 infection and PCR Not Tested cases

***SARS-CoV-2 Seronegative Comparison Group N ~ 4900***

- Healthcare worker at GOSH (other UK and European Hospital Healthcare workers at corresponding sites)
- ≥18 years of age
- ALL have confirmed negative antibodies to SARS-CoV-2 infection on baseline screen

Then 1 of:

**Core Comparison Group N ~ 4200**

## Have not had clinical symptoms consistent with SARS-CoV-2 infection*

OR

**Other Seronegatives N ~ 700**

B) Have had sympoms of SARS-CoV-2 infection but have been tested and were PCR positive or negative but have not developed antibodies at 21 days

*Clinical Symptoms for SARS-CoV-2 infection are defined as one of:

1) New and persistent cough

2) Confirmed temperatures of 37.8 and above

3) Anosmia or altered taste sensation

4) Extreme fatigue

## 8.2 Exclusion Criteria

- <18 years of age
- On immunosuppressive or immunomodulatory medication that may impact test reliability
- Received any blood product including immunoglobulins after September 2019
- Has received convalescent sera as treatment
- Current diagnosis of a malignancy that may impact test reliability
- Those lacking capacity to provide informed consent

**8.3 Study Flow Diagram 1**


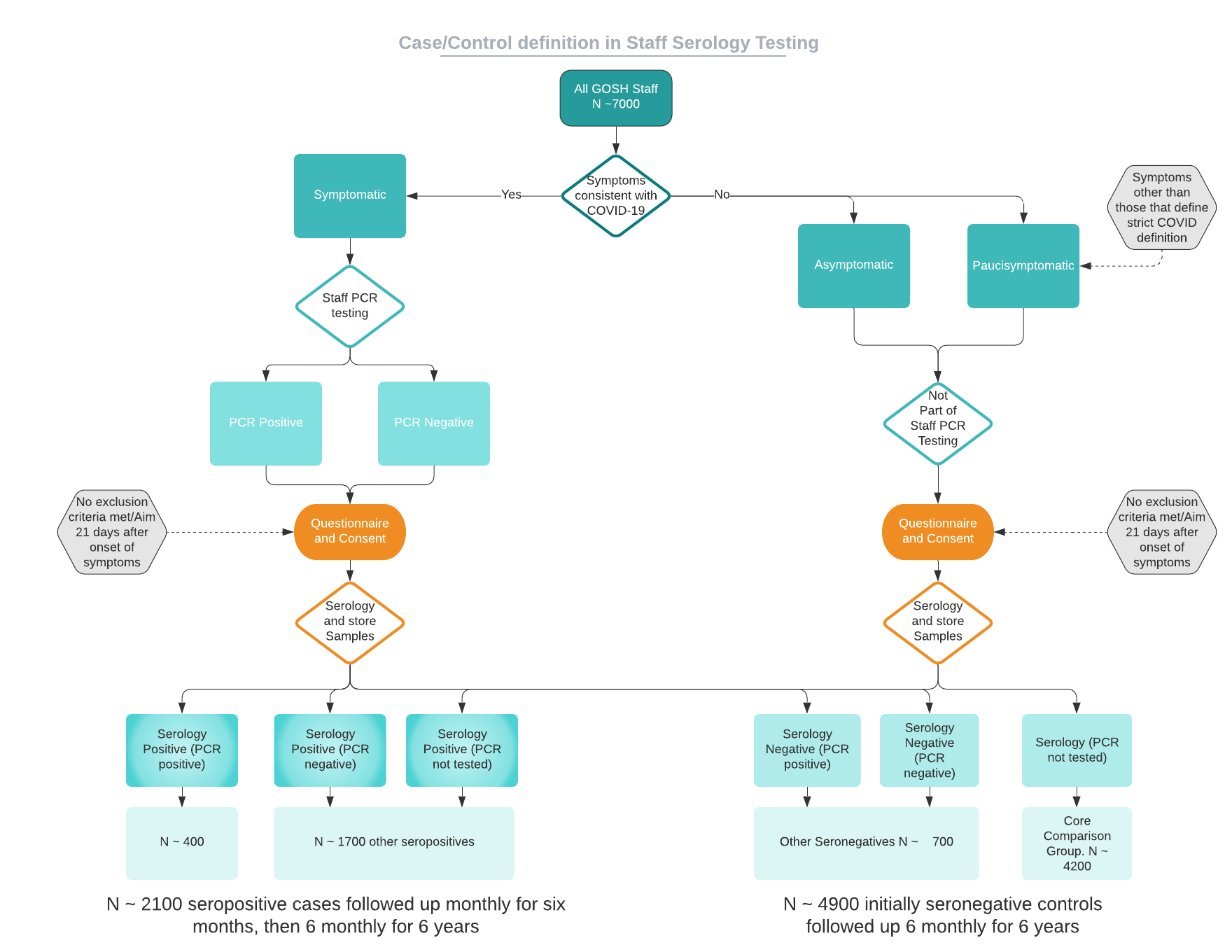


# Study Procedures

## Recruitment

The trust communications team will send an all staff email to inform them about this research project with an invitation link to read the information and eligibility criteria. The microbiology/infection control department at GOSH will also send out a communication to all GOSH staff who have tested positive (or negative) for SARS-CoV-2 as part of the staff testing program to raise awareness of the study and provide the option to join . This approach will permit the dissemination of study to the core study group without study staff having access to identifiable information. We will also consent staff for the use of stored serum and PCR samples taken as part of this staff testing program for use in future research. This component is optional. The positive arm of the study (and negative controls) will have the option of providing a 20ml in EDTA sample for T-cell assay studies, and a salivary sample for IgA analysis, both for research purposes. As these are research tests, results of these extra assays will not be provided to participants. They will be formally consented for this.

Informed consent will then take place in person (or if preferred by telephone or using a zoom meeting). During the informed consent process participants will be provided with information and the chance to answer questions they may have about the study. We will not require the use of any resources or medical records to identify study staff as they are colleagues within the same hospital trust as the study investigators. We will ensure that recruited populations are representative of staff at GOSH (which has a slight majority of women) and that age ranges are representative of the trust population age distribution.

If the participant prefers to undertake a telephone or zoom consent (to maintain physical distancing) we will follow established GOSH trust guidelines. Telephone consent will be witnessed by another member of study staff and co-signed. Study staff undertaking consent will confirm the name and date of birth of the participant without stating their name. At the end of the conversation the witnessing member of staff will speak to the participant to confirm that they have agreed to join the study, then co-sign the document.

After informed consent is completed, participants undertake an online health questionnaire before booking an appointment at the study-run phelobotomy clinic for serological testing. Results of the serological testing will be returned to the participants by secure trust e-mail or via text. We will carefully describe the meaning and significance of a positive, equivocal and negative test in the automated results email as described on the participant information sheet. After returning results, participants will be invited to a follow up appointment in 1 to 2 months or 6 months depending on which arm of the study their results place them in. Early experience established that a small minority of staff (particularly the temporary catering, cleaning and portering staff) do not have trust e-mails. If this is the case, these participants will be asked if they would be happy to receive their serology results over the phone, verbally or by text message; if this is not feasible, we will ask to send their results to their personal email address or to have them collected in person from the study team.

## Informed Consent

Only Good Clinical Practice certified, Human Tissue Act trained, study trained personel who have undertaken the GOSH specific consent training modules and are registered in the study log will be permitted to obtain informed consent. All those who have completed training and are able and eligible to undertake informed consent will be named by the CI/PI on the study log. A detailed information sheet has been prepared (Appendix C: Study Information Sheet). In lay terms this document details the rationale for the study, how and why the participant has been identified and what will happen if potential participants enrol in the study. The information sheet clearly documents the possible inconveniences and benefits of being involved in the study, that enrolling in the study is completely optional and that withdrawl from the study at any stage is also possible at any time.

The secure management of study data is discussed along with the steps taken to minimize the risk of a breach of data security. As this study involves the storage of blood samples for testing and for future work the information sheet explains this in a dedicated section on tissue storage and generic consent for future research. Potential participants will be allowed as much time as they need to decide whether or not they wish to participate in the study.

## Screening and Eligibility Assessment

Screening of potential applicants by study staff will occur when they attend the consent meeting (either face to face, by telephone or by zoom meeting). The process is simple and only involves ensuring that two criteria are met.

1. Either the staff member has had symptoms of possible, probable or confirmed SARS-CoV-2 infection (as defined in this protocol) and must wait for completion of 21 days from symptom onset before entering the study

OR

1. The staff member is asymptomatic but has been exposed to someone with suspected or confirmed SARS-CoV-2 infection and must wait for the completion of 21 days from the start of this exposure
2. The staff member has not had exposure to or symptoms consistent with SARS-CoV-2 infection so can enter the study immediately

Once eligibility has been confirmed, informed consent will be sought and an informed consent document completed and signed off (either face to face by the study participant or, if consent is not taken in person, then by two trained members of the study team). Study participants will then be directed to an online survey (health questionnaire) which they will need to complete to prior to arranging an appointment with the phlebotomy team for blood sampling.

All staff at GOSH will also be asked if they would like to be made aware of other parallel COVID related studies running at GOSH and UCL. If they express interest in this then they will be directed to the relevant study websites/email addresses.

## Baseline Assessments

Baseline assessments will include the completion of a standardized questionnaire (Appendix D: Study Standardized Questionnaire) followed by an appointment for a blood test to be taken by trained study staff. At this appointment we will take 4ml of blood for serological testing and 4ml of EDTA blood for storage and future research.

We have amended the online survey to include details about other emerging SARS-CoV-2 risk factors including medical conditions, ethinic background (BAME), home occupancy and ability to distance in the workplace. We have also added a second email address box that allows to follow up staff once they have left the trust, we have expanded the previous testing history to include additional information about home testing kits and other ELISA tests. Severe disease markers and complications of COVID-19 are also included and vaccination history is also included to prepare for when vaccination is rolled out.

Questionnaire development will utilise technology similar to other NHS staff surveys that allow completion on personal mobile devices. The questionnaire interface would be separated from the database, so they can employ different levels and methods of security. Many hosting services have built-in defences like firewalls, access logs, and authorisation protocols to restrict access, and the stored data should also be encrypted using a strong algorithm. Any data transferred to the database, e.g. via the interface, should also be encrypted to ensure that it is unreadable to anyone who intercepts it. If the database is a SQL database, the interface must also employ defences against "SQL injection" attacks, to prevent any code entered into forms doing any damage or exposing the data.
Regularly migrating identifiable information to internal servers (perhaps while leaving some anonymised statistical data if this is required) would also significantly reduce the risk of data theft.

## Subsequent Visits

Subsequent follow-up visits will be explained to all potential study participants as part of the informed consent process. The 2100 seropositive serology cohort will be followed intensively every 1 to 2 months for repeated serological testing whilst the 4900 seronegative comparison group will be followed 6 monthly. Participants from the positive arm of the study (as well as negative controls) will have the option of providing a 20ml in EDTA sample for T-cell assay studies and tissue typing, and a salivary sample for IgA analysis, both for research purposes. As these extra assays are research tests, results will not be provided to particpants. They will be formally consented for this.

After the initial intensive phase, all ~7000 recruits will be followed 6 monthly for the 6 year duration of the study. Follow-up visits will be arranged following the baseline assessment. The same blood tests 4ml EDTA and 4ml serum will be taken at each follow up appointment as well as the same symptom questionnaire. If a COVID-19 vaccination becomes available during the study we will undertake performance evaluation of an ELISA suitable for monitoring antibody titres to the vaccine target. We will then continue to follow up our cohort with the additional focus on the antibody titres to vaccination, duration of immunity following vaccination and the attack rate in the vaccinated and unvaccinated groups (with the previso that we expect the vaccine will have wide and rapid uptake).

Further detailed questionnaires will be sent to subgroups found to have higher incidence of infection, to further delineate the susceptibility and exposure factors.

## Study Duration

The study will run for 6 years and 3 months.

## Discontinuation/Withdrawal of Participants from Study

Although we anticipate a high uptake, engagement and retention with the study due to the common first hand experience with SARS-CoV-2, participants may withdraw from the study at any stage. The information sheet clearly states the email addresses of the study investigators that can be contacted should the participant wish to withdraw from the study. No further procedures or observations will be required. Participants will have the option of leaving the study but allowing data to be kept and samples stored for research OR leaving the study, erasing all data and destroying all samples. A note will be made on the study record and the "last successful follow-up" date will be taken as the "lost to follow-up/left the study" date for the purposes of statistical analysis. Whilst the data from previous questionnaires is still able to be identified, if the study participant requests that all their data be erased or removed from the database, it will be excluded from analysis.

## Definition of End of Study

The study will end on the final follow up visit of the final recruit 6 years after the final recruit joined the study. As we aim to recruit the first ~7000 healthcare workers within the first 3 months of the study we plan to stop the study at 6 years and 3 months after starting the study. We will undertake interim analyses at the end of each year. In the unlikely scenario of all participants having undetectable antibodies we will end the study early.

# Intervention

The only interventions undertaken in this study are standardized quesionnaires (Appendix D) and blood tests (8ml) to measure the antibody titres to the SARS-CoV-2 specific protein (4ml) and 4ml EDTA to be stored for future research. These will be undertaken at each pre-planned follow up as per our flow diagrams (Appendix A). Participants will also be consented for an additional blood test of 20ml to study SARS-CoV-2 T-cell immunity and salivary sample for IgA. These optional consents will detail that samples will be tissue typed.

# Subject Withdrawal Criteria

The choice of whether to withdraw from the study is dependent on the participant. As clearly explained in the information sheet they are free to withdraw from the study at any time. For those that withdraw from the study they may choose to a) leave the study but leave all their data and samples in order to contribute to the research project or b) erase the data and destroy the samples. In the latter case clearly these participants will not be able to be considered in the final analysis. If participants choose to leave the study, but agree to the usage of their data up until the point of leaving we will consider them "lost to follow-up" at their final successful follow-up interview and serological test. We currently do not have the resources to replace subjects that withdraw from the study but should funding support be obtained we would then do so. Equally should funding allow support to recruit more patients to improve the power and reduce the detectable effect size we will do so. If full funding is obtained for the project we will aim to scale up recruit all staff members at the trust.

# Statistics

## Statistical methods to be employed (plan of analysis)

Antibody titres are expected to follow a negative exponential (log-linear) decay over time as seen following other respiratory viral infections. Serial antibody titres will be log-transformed and then multivariate linear regression models will be used to determine the predictors of antibody decay. Variable attack-rate between groups will be compared with a two-sample test of proportions and the incidence of COVID-19 disease between study groups will be compared using survival analysis with log-rank testing.

Fig.1: An estimated sample size of 4500-8000 total participants (with seropositive sample size of 1500-2700 participants, Fig.1, x-axis) provides 80% power at alpha 0.05 to detect an effect size of ≥17% decline in antibody titres over 12-months. This is based on a seropositivity prevalence of 33%, as observed in preliminary results so far and a negative exponential (log-linear) model of waning antibody titres, with 3 co-variate predictors of antibody decay, including sex, age and ethnicity (green line) or 4 co-variate predictors, including sex, age, ethnicity and co-morbidities (blue line). For comparison, the model was repeated based on 20% decline in baseline antibody titres over 12-months (using alpha 0.05), with the same 3 (orange line) or 4 (red line) co-variate predictors of antibody decline.


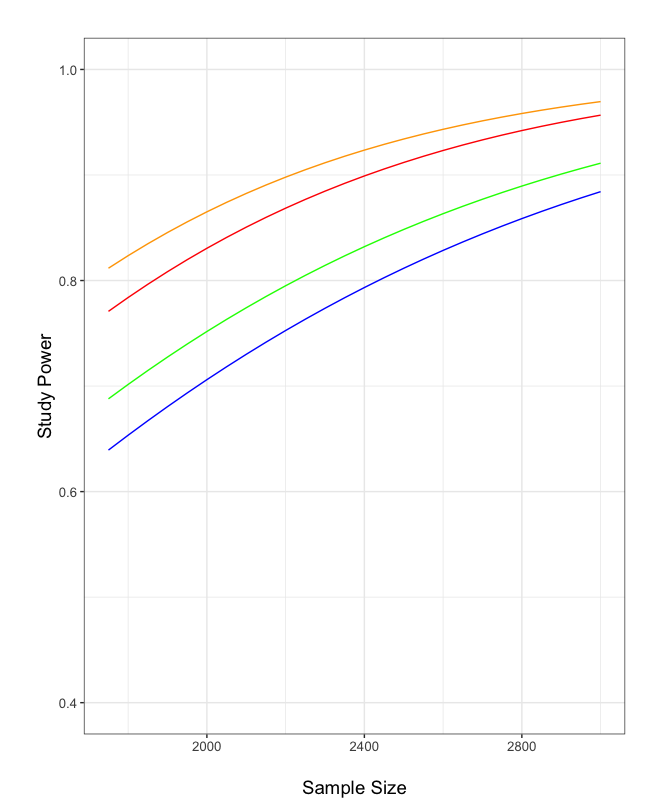


In order to determine the study power for varying effect sizes with unequal group sizes we undertook a two-sample power calculation of proportions with group sizes of 2100 seropositive cases and 4900 seronegative comparators using R (foundation for statistical computing) pwr function pwr.2p2n.test (Figure 2).

Figure 2. Power curve for group sizes of 2100 and 4900 participants showing that this study will have the power to detect an effect size of 0.07 i.e. a difference in the proportion in the attack rate between the two groups of 7%.


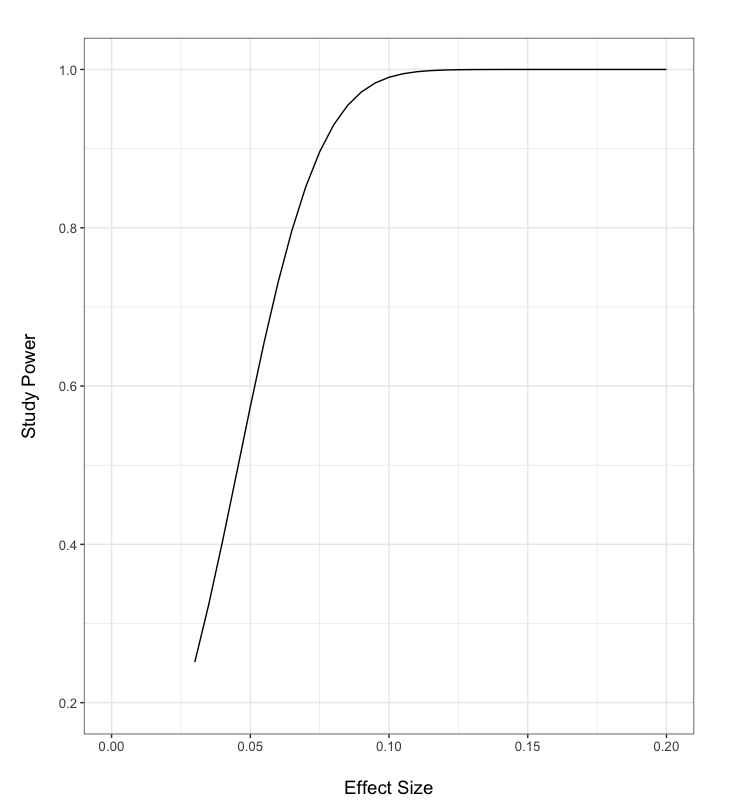


We will also undertake an exploratory analysis using survival analysis to compare the hazards of infection in both groups over the course of the study using the date of reported and PCR proven SARS-CoV-2 infection as an event and the last follow up date or the final interview as the censorship date. We anticipate a high retainment rate in the study for three reasons a) GOSH has high baseline staff retention rates b) hospital staff already understand the rationale and the importance of this research and c) we have already received high levels of interest and engagement with the study.

# Data Management

## Source Documents

The study will not require access to any of the participants medical records as part of this research. Instead new recruits will be asked to help complete an online questionnaire about their health, symptoms and exposure to SARS-CoV-2. Systems already employed by Occupational Health at GOSH have been duplicated to allow data collection of on online questionnaires on GOSH secure servers. This process has an established system of governance approved by GOSH ICT department. No hard copies of the identifiable data will be downloaded. The laboratory data will be stored as for existing occupational health medical information (such as Hepatitis B serology) which are stored on a secure NHS trust laboratory server. Samples will be stored in -70C freezers with a laboratory number unliked from any patient identifiers. Only named study staff will have access to the data. On all study-specific documents, other than the signed consent, the participant will be referred to by the study participant number/code, not by name.

## Direct Access to source data / documents

Only members of the study research team and authorised representatives from the sponsor will have direct access to the source data and study documentation. All source data and study documentation will also be available to external auditors if and when required, and inspections in the event of a regulatory inspection. Access to the final data set will remain with the chief investigator and co-investigators.

## Data Recording and Record Keeping

After providing informed consent, participants will be provided with a link to an online study questionnaire. This will be stored on GOSH secure servers and completed prior to staff phlebotomy appointment. Only named study staff will have access to the raw data. As soon as possible after collection identifiable data will be stored in a separate database to medical data. The data will be linked only by a study ID to which only named study staff will have access to. Data will be retained for the 6 year duration of the study after which it will remain pseudonymized for internal use and future research and completely anonymized for sharing with research collaborators or publication.

## Archiving

Archiving will be authorised by the Sponsor following submission of the end of study report. We will also submit the complete but anonymized dataset to a secure and publicly available database such as Dryad (used by PLOS Medicine) to enable access to other researchers. Essential study documents will be retained for a 30 years after completion of the study. These documents will be retained for longer if required by the applicable regulatory requirements.

# Patient Confidentiality & Data Protection

Participant identifiable data, including name, date of birth, email and telephone number will be required for the registration process. Entering all data to an online database on a secure trust server will ensure the highest level of security available to minimize the risk of a breach in confidentiality. Identifiable data will be stored with a study ID separately to a linked database with medical data. All documents will be stored securely and only accessible by study staff and authorised personnel. The study will comply with the Data Protection Act 2018, which requires data to be anonymised as soon as it is practical to do so at the end of the study.

# Sample Collection, Storage, Transfer and Analysis

Blood samples will be collected by trained phlebotomists, nursing staff, doctors or medical students. Once informed consent has been provided and the online questionnaire is complete, appointments will be made with the study phlebotomy clinic. The phlebotomy clinic will be initially located in the outpatients clinic area as it is currently vacant due to the pandemic. When outpatient appointments resume, we will resume testing in a designated part of the hospital with equivalent facilities. Each appointment will be 15-20 minutes long in order to prevent unnecessary mixing between staff. Using an aseptic technique with alcowipes, a vacutainer system, a 21 guage green needle and a tourniquet, a 4ml sample of blood will be taken in a BD Vacutainer Serum Separating Tube (SST) Advance Tube and a 4ml sample will be taken in an BD Vacutainer EDTA tube. For seropositive and equivocal participants who further consent, an additional 20ml blood sample will be taken for T-cell assay and tissue typing, in EDTA tubes and approximately 4ml of a salivary sample for IgA assay. For blood samples a cotton wool ball will be used to prevent bleeding and 1cm circular plaster applied. For whole saliva samples, participants will be asked to passive drool (the gold standard sample), unstimulated, into polypropylene cryovials. The samples will be transported in a sealed specimen transfer box to the Camelia Botnar laboratory at Great Ormond Street in batches twice per day and be stored at -4^o^C in restricted access fridges until sample processing. Those samples arriving in the laboratory in the late afternoon will be processed the following morning, while those samples arriving from the morning batch will be processed the same day. All samples will be given a laboratory code on arrival and entered into the secure Beaker system database under this laboratory code. It is possible to batch up to 45 samples in duplicate on each run, using our DS2 automated ELISA processing system from Dynex Technologies. Each sample will be tested using the *EDI^TM^* diagnostic assay which detects the optical density of antibodies in serum samples to the specific protein SARS-CoV-2. IgG antibodies to the specific protein will be tested and reported. As and when new ELISA assays with superior performance become available, we will test them using our sample bank of positive and negative controls before considering replacing our current gold standard assay. This applies particularly to ensuring study alignment in our collaboration with the SIREN study at Imperial College who use different serological assays. Neutralization assays will also be undertaken in appropriate P3 biosafety laminar flow cabinets in order to determine the in-vitro correlates of antibody protection. Cell mediated immune responses to purified spike protein will also be tested for study participants using ELISPOT or flow cytometry. This will enable us to determine the titres of antibody associated with a 50% reduction in viral plaques and the relevance of cell mediated immunity on future reinfection. This work will be lead by Dr Kimberly Gilmour and Professor Goldblatt who both have 20 years experience of developing, evaluating and standardizing new immunological assays in the laboratory. In order to improve capacity of antibody testing for this study, the WHO Reference Laboratory for Pneumococcal Serology, a GCLP accredited laboratory  run by Professor David Goldblatt and based in the Great Ormond Street Institute of Child Health will be used. A panel of +ve and -ve samples have been assessed in both laboratories with excellent concordance between the laboratories. Samples labelled with the unique study ID only will be handled in the UCL laboratory and results, on a spreadsheet, will be returned to the GOSH laboratory where the results will be entered into the master database. NO DATA will be transferred from GOSH to UCL. When new assays become available they will also be evaluated in the UCL laboratory with the same arrangements for sample and data transfer.

At the end of the study the samples will be stored at -70^o^C in the freezers of the Great Ormond Street Camelia Botnar Laboratories for future research. Future studies may involve genetic testing on the samples and this has been highlighted on the consent form. However independent ethical, institutional and regulatory approval will have to be sought before those studies and access to the samples are permitted. The consent form clearly states that staff may need to be asked to re-consent in the future before future genetic testing. We would also like to use the collected samples in the future to improve our diagnostic test and compare it to other tests that are developed over time and may be better.

The study will not share samples with collaborators that are linked with any identifiable data, although we may use non-identifiable samples to collaborate with international colleagues to enabled improved testing of SARS-CoV-2. This will be done once ethical, institutional and regulatory approvals are in place and under appropriate agreements as necessary.

# Financial Information and Insurance

Funding has been provided by GOSH to test the first 1000-1250 staff members and undertake the baseline assessment. However we are actively seeking funding to allow for this cohort to be followed up over time. As this study is non-interventional observational prospective cohort study we do not anticipate any adverse events other than those associated with a blood test. As with any research undertaken at GOSH and UCL ICH, cover for negligent harm will be provided by the Great Ormond Street Hospital for Children NHS Foundation Trust through the Clinical Negligent Scheme for Trusts (CNST). No-fault compensation insurance cover for any non-negligent harm will be provided by University College London.

# Publications Policy

All individuals who have made substantial intellectual, scientific and practical contributions to the study and the manuscript will be credited as authors and this will be overseen by the Chief Investigator, co-PI's and the Sponsors. In all cases where journal policies permit, all investigators who contribute patients to the study will be acknowledged.

The results of the study will be published in open access peer-reviewed scientific journals. Internal reports will be published on the UCL Institute of Child Health Web Pages which ware publicly assessible. We will also present our study findings at ECCMID and provide writted feedback of the study recruits by email.

# References

1. Huang C, Wang Y, Li X, et al. Clinical features of patients infected with 2019 novel coronavirus in Wuhan, China. *Lancet*. 2020;395(10223):497-506. doi:10.1016/S0140-6736(20)30183-5

2. Johns Hopkins University. Coronavirus COVID-19 Global Cases by the Center for Systems Science and Engineering (CSSE) at Johns Hopkins University (JHU). https://gisanddata.maps.arcgis.com/apps/opsdashboard/index.html#/bda7594740fd40299423467b48e9ecf6. Accessed April 10, 2020.

3. Zhu N, Zhang D, Wang W, et al. A novel coronavirus from patients with pneumonia in China, 2019. *N Engl J Med*. 2020;382(8):727-733. doi:10.1056/NEJMoa2001017

4. Zhou P, Yang X Lou, Wang XG, et al. A pneumonia outbreak associated with a new coronavirus of probable bat origin. *Nature*. 2020;579(7798):270-273. doi:10.1038/s41586-020-2012-7

5. Lu R, Zhao X, Li J, et al. Genomic characterisation and epidemiology of 2019 novel coronavirus: implications for virus origins and receptor binding. *Lancet*. 2020;395(10224):565-574. doi:10.1016/S0140-6736(20)30251-8

6. Aylward, Bruce (WHO); Liang W (PRC). Report of the WHO-China Joint Mission on Coronavirus Disease 2019 (COVID-19). *WHO-China Jt Mission Coronavirus Dis 2019*. 2020;2019(February):16-24. https://www.who.int/docs/default-source/coronaviruse/who-china-joint-mission-on-covid-19-final-report.pdf.

7. Guan W, Ni Z, Hu Y, et al. Clinical Characteristics of Coronavirus Disease 2019 in China. *N Engl J Med*. February 2020. doi:10.1056/nejmoa2002032

8. Zhao S, Lin Q, Ran J, et al. Preliminary estimation of the basic reproduction number of novel coronavirus (2019-nCoV) in China, from 2019 to 2020: A data-driven analysis in the early phase of the outbreak. *Int J Infect Dis*. 2020;92:214-217. doi:10.1016/j.ijid.2020.01.050

9. To KK-W, Tsang OT-Y, Leung W-S, et al. Temporal profiles of viral load in posterior oropharyngeal saliva samples and serum antibody responses during infection by SARS-CoV-2: an observational cohort study. *Lancet Infect Dis*. 2020;0(0). doi:10.1016/s1473-3099(20)30196-1

10. Zhang J, Wang S, Xue Y. Fecal specimen diagnosis 2019 novel coronavirus–infected pneumonia. *J Med Virol*. March 2020:jmv.25742. doi:10.1002/jmv.25742

11. Water Transmission and COVID-19: Questions and Answers. https://www.cdc.gov/coronavirus/2019-ncov/php/water.html. Accessed April 10, 2020.

12. Weiss SR, Leibowitz JL. Coronavirus pathogenesis. In: *Advances in Virus Research*. Vol 81. Academic Press Inc.; 2011:85-164. doi:10.1016/B978-0-12-385885-6.00009-2

13. Xie M, Chen Q. Insight into 2019 novel coronavirus — an updated intrim review and lessons from SARS-CoV and MERS-CoV. *Int J Infect Dis*. April 2020. doi:10.1016/j.ijid.2020.03.071

14. Lauer SA, Grantz KH, Bi Q, et al. The Incubation Period of Coronavirus Disease 2019 (COVID-19) From Publicly Reported Confirmed Cases: Estimation and Application. *Ann Intern Med*. March 2020. doi:10.7326/m20-0504

15. Lou B, Li T-D, Zheng S-F, et al. Serology characteristics of SARS-CoV-2 infection since the exposure and post symptoms onset. doi:10.1101/2020.03.23.20041707

16. Gane SB, Kelly C, Hopkins C. Isolated sudden onset anosmia in COVID-19 infection. A novel syndrome? *Rhinology*. April 2020. doi:10.4193/Rhin20.114

17. *Loss of Sense of Smell as Marker of COVID-19 Infection*.

18. Xie J, Tong Z, Guan X, Du B, Qiu H, Slutsky AS. Critical care crisis and some recommendations during the COVID-19 epidemic in China. *Intensive Care Med*. 2020:1. doi:10.1007/s00134-020-05979-7

19. Xu Z, Shi L, Wang Y, et al. Pathological findings of COVID-19 associated with acute respiratory distress syndrome. *Lancet Respir Med*. 2020;8(4):420-422. doi:10.1016/S2213-2600(20)30076-X

20. Xie J, Tong Z, Guan X, Du B, Qiu H. Clinical Characteristics of Patients Who Died of Coronavirus Disease 2019 in China. *JAMA Netw Open*. 2020;3(4):e205619. doi:10.1001/jamanetworkopen.2020.5619

21. Garg S, Kim L, Whitaker M, et al. Hospitalization Rates and Characteristics of Patients Hospitalized with Laboratory-Confirmed Coronavirus Disease 2019 — COVID-NET, 14 States, March 1–30, 2020. *MMWR Morb Mortal Wkly Rep*. 2020;69(15). doi:10.15585/mmwr.mm6915e3

22. Mizumoto K, Kagaya K, Zarebski A, Chowell G. Estimating the asymptomatic proportion of coronavirus disease 2019 (COVID-19) cases on board the Diamond Princess cruise ship, Yokohama, Japan, 2020. *Euro Surveill*. 2020;25(10). doi:10.2807/1560-7917.ES.2020.25.10.2000180

23. Day M. Covid-19: four fifths of cases are asymptomatic, China figures indicate. *BMJ*. 2020;369:m1375. doi:10.1136/bmj.m1375

24. Tang Y-W, Schmitz JE, Persing DH, Stratton CW. The Laboratory Diagnosis of COVID-19 Infection: Current Issues and Challenges 1 2 3 Downloaded from. *J Clin Microbiol*. 2020. doi:10.1128/JCM.00512-20

25. Fang Y, Zhang H, Xie J, et al. Sensitivity of Chest CT for COVID-19: Comparison to RT-PCR. *Radiology*. February 2020:200432. doi:10.1148/radiol.2020200432

26. West CP, Montori VM, Sampathkumar P. Journal Pre-proof COVID-19 Testing: The Threat of False-Negative Results. *JMCP*. 2020. doi:10.1016/j.mayocp.2020.04.004

27. Zhang W, Du RH, Li B, et al. Molecular and serological investigation of 2019-nCoV infected patients: implication of multiple shedding routes. *Emerg Microbes Infect*. 2020;9(1):386-389. doi:10.1080/22221751.2020.1729071

28. Xiang J, Yan M, Li H, et al. Evaluation of Enzyme-Linked Immunoassay and Colloidal Gold- Immunochromatographic Assay Kit for Detection of Novel Coronavirus (SARS-Cov-2) Causing an Outbreak of Pneumonia (COVID-19). *medRxiv*. March 2020:2020.02.27.20028787. doi:10.1101/2020.02.27.20028787

29. Lin D, Liu L, Zhang M, et al. Evaluations of serological test in the diagnosis of 2019 novel coronavirus (SARS-CoV-2) infections during the COVID-19 outbreak. *medRxiv*. March 2020:2020.03.27.20045153. doi:10.1101/2020.03.27.20045153

30. Liu R, Liu X, Han H, et al. The comparative superiority of IgM-IgG antibody test to real-time reverse transcriptase PCR detection for SARS-CoV-2 infection diagnosis. *medRxiv*. 2020:2020.03.28.20045765. doi:10.1101/2020.03.28.20045765

31. Zhang B, Zhou X, Zhu C, et al. Immune phenotyping based on neutrophil-to-lymphocyte ratio and IgG predicts disease severity and outcome for patients with COVID-19. doi:10.1101/2020.03.12.20035048

32. Antibody responses to SARS-CoV-2 in patients of novel coronavirus disease 2019 | Clinical Infectious Diseases | Oxford Academic. https://academic.oup.com/cid/advance-article/doi/10.1093/cid/ciaa344/5812996. Accessed April 12, 2020.

33. WHO. *2019 Novel Coronavirus: Overview of the State of the Art and Outline of Key Knowledge Gaps A Coordinated Global Research Roadmap*. https://www.who.int/blueprint/priority-diseases/key-action/Coronavirus_Roadmap_V9.pdf?ua=1. Accessed April 10, 2020.

34. Global Progress on COVID-19 Serology-Based Testing. http://www.centerforhealthsecurity.org/resources/COVID-19/serology/Serology-based-tests-for-COVID-19.html. Accessed April 11, 2020.

35. Wu LP, Wang NC, Chang YH, et al. Duration of antibody responses after severe acute respiratory syndrome. *Emerg Infect Dis*. 2007;13(10):1562-1564. doi:10.3201/eid1310.070576

36. MO H, ZENG G, REN X, et al. Longitudinal profile of antibodies against SARS-coronavirus in SARS patients and their clinical significance. *Respirology*. 2006;11(1):49-53. doi:10.1111/j.1440-1843.2006.00783.x

37. Wang H-J, Zhang L-L, Tan W-J, et al. [Consecutive five-year follow-up analysis of specific IgG antibody of 22 cases of SARS patients after recovery]. *Bing du xue bao = Chinese J Virol*. 2010;26(4):295-297. http://www.ncbi.nlm.nih.gov/pubmed/20836383. Accessed April 11, 2020.

38. Guo X, Guo Z, Duan C, et al. Long-Term Persistence of IgG Antibodies in SARS-CoV Infected Healthcare Workers. *Medrxiv*. February 2020:2020.02.12.20021386. doi:10.1101/2020.02.12.20021386

39. Petherick A. *Developing Antibody Tests for SARS-CoV-2*. Vol 395.; 2020. doi:10.1016/S0140-6736(20)30788-1

40. Payne DC, Iblan I, Rha B, et al. Persistence of antibodies against middle east respiratory syndrome coronavirus. *Emerg Infect Dis*. 2016;22(10):1824-1826. doi:10.3201/eid2210.160706

41. Lee N, Chan PKS, Ip M, et al. Anti-SARS-CoV IgG response in relation to disease severity of severe acute respiratory syndrome. *J Clin Virol*. 2006;35(2):179-184. doi:10.1016/j.jcv.2005.07.005

42. Zhang L, Zhang F, Yu W, et al. Antibody responses against SARS coronavirus are correlated with disease outcome of infected individuals. *J Med Virol*. 2006;78(1):1-8. doi:10.1002/jmv.20499

43. Bao L, Deng W, Gao H, et al. Reinfection could not occur in SARS-CoV-2 infected rhesus macaques 2 3 Beijing Key Laboratory for Animal Models of Emerging and Remerging Infectious. doi:10.1101/2020.03.13.990226

44. Yuen KS, Ye ZW, Fung SY, Chan CP, Jin DY. SARS-CoV-2 and COVID-19: The most important research questions. *Cell Biosci*. 2020;10(1). doi:10.1186/s13578-020-00404-4

45. Shi Y, Wang Y, Shao C, et al. COVID-19 infection: the perspectives on immune responses. *Cell Death Differ*. March 2020:1-4. doi:10.1038/s41418-020-0530-3

46. Peeples L. News Feature: Avoiding pitfalls in the pursuit of a COVID-19 vaccine. *Proc Natl Acad Sci U S A*. March 2020. doi:10.1073/pnas.2005456117

47. Liu Y, Michelson D, Clark R, Gold JA. Child Neurology: Siblings with infantile epilepsy and developmental delay: A circuitous path to genomic diagnosis. *Neurology*. 2018;91(3):143-147. doi:10.1212/WNL.0000000000005815

# 19 Appendix A: Study Flow Charts


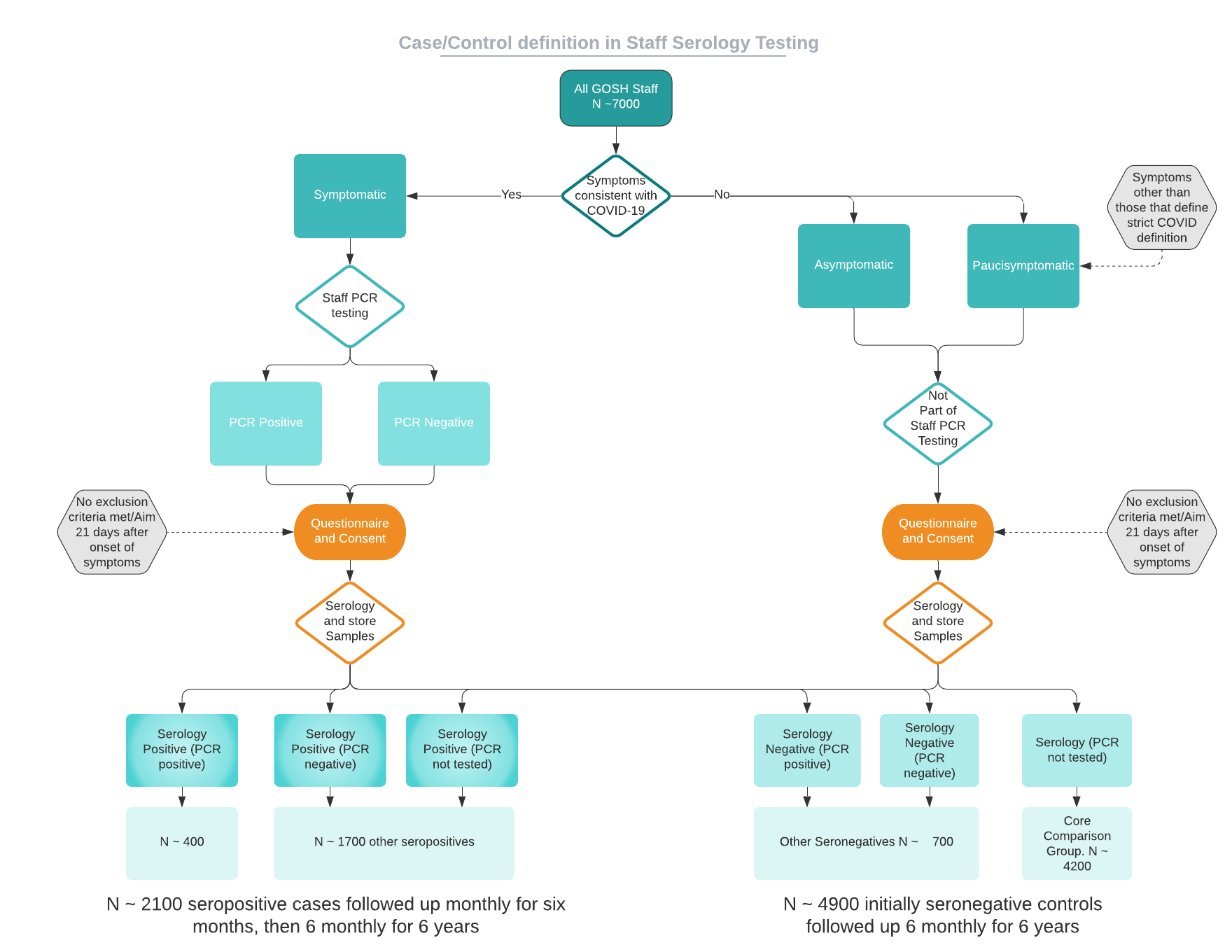

# Appendix B: Schedule of Procedures

| **Procedures** | **Baseline Screening, Baseline Blood Tests and Follow-Up** | | | |
| --- | --- | --- | --- | --- |
|  | **Screening** | **Baseline Blood Tests** | **Follow-Up N=200 Cases (x18)** | **Follow-Up N=800 Comparison Group (x13)** |
| Informed consent | X |  | X | X |
| Demographics | X |  | X | X |
| Medical history | X |  | X | X |
| Concomitant medications | X |  | X | X |
| Physical examination |  |  |  |  |
| ECG |  |  |  |  |
| Laboratory tests |  | X | X | X |
| Eligibility assessment | X |  |  |  |
| Randomisation |  |  |  |  |
| Intervention |  |  |  |  |

**21 Appendix C: Questionnaire**

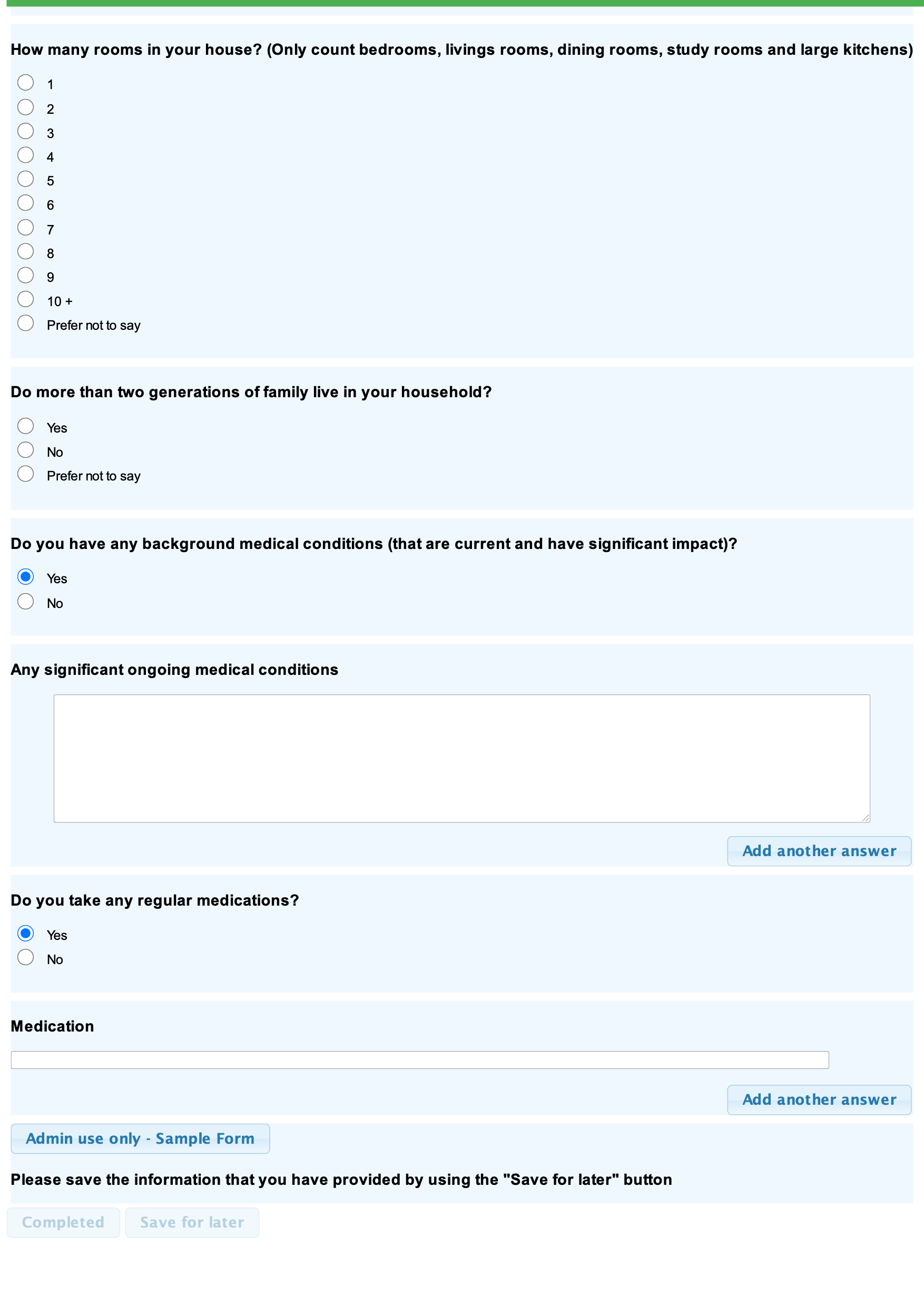


**22 Appendix D: Sites of collaboration**

**International:**

| **Site No.** | **Name/Lead** | **Institution** | **Country** |
| --- | --- | --- | --- |
| 1 | Dace Zavadsaka | Riga Stradina Universitate, Vienibas gatve 45, Riga, LV-1004 | Latvia |
| 2 | Inga Ivaskeviciene | Vilnius University Hospital Santaros Klinikos, Vilnius Lithuania | Lithuania |
| 3 | Eda Tamm | Tartu University Hospital, Ludvig Puusepa 1a, 50406 Tartu | Estonia |
| 4 | Ásgeir Haraldsson | Children's Hospital Iceland, Landspitali University Hospital, 101-Reykjavik | Iceland |
| 5 | Oana Falup-Pecurariu | Children's Clinic Hospital, Transilvania University, Brasov, | Romania |
| 6 | Vana Spoulou | Dept of Paediatrics, “Agia Sophia” Children’s Hospital of Athens, Τhivon & M.Asias 1, Goudi 11527, Athens | Greece |
| 7 | Heather Zar | Red Cross Children's Hospital, Cape Town | South Africa |
| 8 | Dr. Michael Wagner | Medizinische Universität Wien,  Comprehensive Center for Pediatrics, Währinger Gürtel 18-20, 1090 Vienna | Austria |
| 9 | Tea Nieminen | Department of Pediatrics, Helsinki University Central Hospital,  Helsinki | Finland |
| 10 | Pere Soler Palacin | Hospital Universitari Vall d’Hebron, Vall d’Hebron Barcelona Hospital Campus, Barcelona | Spain |
| 11 | Ronan Leahy | Childrens Health Ireland at Crumlin, National Children's Research Centre, Dublin | Ireland |
| 12 | Olivier Van Den Berg | Pôle Hospitalier Universitaire de Bruxelles (PHUB), Brussels, Belgium | Belgium |

**National/UK**

|  | **Name/Lead** | **Institution** | **Country** |
| --- | --- | --- | --- |
| 1 | Matthias Koepp | Chalfont Center, UCLH, London | U.K. |
| 2 | Suzannah Felsenstein | Alderhey Hospital, Alderhey | U.K. |
| 3 | Susan Hopkins | Imperial NHS Trust, London | U.K. |
